# Atom interaction propensities of oxygenated chemical functions in crystal packings

**DOI:** 10.1107/S2052252516020200

**Published:** 2017-01-21

**Authors:** Christian Jelsch, Yvon Bibila Mayaya Bisseyou

**Affiliations:** a CRM2, UNR CNRS 7036, Institut Jean Barriol, Université de Lorraine, Vandoeuvre les Nancy CEDEX, France; b Laboratoire de Cristallographie et Physique Moléculaire, UFR SSMT, Université Félix Houphouët-Boigny, 22 BP 582 Abidjan 22, Côte d’Ivoire, France

**Keywords:** hydrogen bonds, halogen bonding, synthons, crystal engineering, enrichment of contacts, statistical analysis, Hirshfeld surface

## Abstract

The crystal contacts of several families of hydrocarbon compounds substituted with one or several types of oxygenated chemical groups were analyzed statistically using the Hirshfeld surface methodology. The propensity of contacts to occur between two chemical types is described with the contact enrichment descriptor. The systematic large enrichment ratios of some interactions like the O—H⋯O hydrogen bonds suggests that these contacts are a driving force in the crystal packing formation. The same statement holds for the weaker C—H⋯O hydrogen bonds in ethers, esters and ketones, in the absence of polar H atoms. The over-represented contacts in crystals of oxygenated hydrocarbons are generally of two types: electrostatic attractions (hydrogen bonds) and hydrophobic interactions. While Cl⋯O interactions are generally avoided, in a minority of chloro-oxygenated hydrocarbons, significant halogen bonding does occur. General tendencies can often be derived for many contact types, but outlier compounds are instructive as they display peculiar or rare features. The methodology also allows the detection of outliers which can be structures with errors. For instance, a significant number of hydroxylated molecules displaying over-represented non-favorable oxygen–oxygen contacts turned out to have wrongly oriented hydroxyl groups. Beyond crystal packings with a single molecule in the asymmetric unit, the behavior of water in monohydrate compounds and of crystals with *Z*′ = 2 (dimers) are also investigated. It was found in several cases that, in the presence of several oxygenated chemical groups, cross-interactions between different chemical groups (*e.g.* water/alcohols; alcohols/phenols) are often favored in the crystal packings. While some trends in accordance with common chemical principles are retrieved, some unexpected results can however appear. For example, in crystals of alcohol–phenol compounds, the strong O—H⋯O hydrogen bonds between two phenol groups turn out to be extremely rare, while cross contacts between phenols and alcohols have enriched occurrences.

## Abstract 2

Intermolecular crystal contacts formed by different oxygen atom types are investigated.

## 1. Introduction   

Finding reliable methods of predicting the crystal structure of a compound, based only on its molecular structure, has been a goal of physical sciences for several decades (Desiraju, 2002 ▸). The prediction of a crystal packing for an organic compound is still difficult and computations costly, although several software now propose some solutions (Thakur *et al.*, 2015 ▸). A recent study on intermolecular energy computation between dimers in the crystal (Turner *et al.*, 2015 ▸) is a deep approach to understanding crystal packings. The Pixel method described by Gavezzotti (2005 ▸) allows for a separation between Coulombic, dispersion, polarization and repulsion-energy contributions in intermolecular contacts. Therefore, their relative importance can be analyzed with respect to the chemical constitution of the interacting partners. Intermolecular interactions control the crystal packing of organic molecules. As a consequence, the accurate calculation of lattice energies in the later stages of crystal-structure predictions is crucial. Price (2004 ▸) has for instance developed non-empirical anisotropic polarizability-based atom–atom potentials for use in crystal-structure prediction studies.

Statistical rules concerning atom–atom contacts in crystals is experimental database derived knowledge which contributes towards a better understanding of crystal packing formation. The forces acting between molecules/functional groups are of electrostatic and van der Waals nature, while interactions are also classified as hydrophobic *versus* hydrophilic.

There is a large amount of literature investigating the role of O atoms in crystal interactions and crystal engineering. Organic Cl, Br and I atoms are considered as hydrophobic. O atoms whether forming two single bonds or one double bond generally bear two lone pairs and are considered to have a negative partial charge. Depending on their chemical environment, notably the presence of electron withdrawing/donating groups, O atoms are more or less electronegative. O atoms bound to a more electropositive H atom such as in alcohols, phenol or carboxylic acids are hydrophilic as they are good hydrogen-bond acceptors. O atoms are particularly important in crystals of organic molecules for their ability to form strong hydrogen bonds (notably O—H⋯O and N—H⋯O). On the contrary, O atoms bound to two C atoms such as in ethers and esters are considered much less hydrophilic and form weaker hydrogen bonds. C—H⋯O contacts have been described as weak hydrogen bonds (Desiraju, 1996 ▸) and a strength hierarchy among weak hydrogen-bond donors and acceptors has been proposed by Steiner (1998 ▸). According to Gilli *et al.* (2009 ▸) weak hydrogen bonds are basically electrostatic, while stronger ones are mixtures of electrostatic and covalent contributions.

The involvement of functional groups in crystal packing contacts is widely investigated in the literature in order to better understand the principles of crystal formation. Galek *et al.* (2010 ▸) have for instance developed the Logit hydrogen-bonding propensity method to accurately predict which hydrogen bonds might form in an organic crystal structure. Hydrogen-bond propensity models were constructed using crystal structures archived in the Cambridge Structural Database (CSD; Allen, 2002 ▸)

Analysis of intermolecular interactions using the Hirshfeld surface-based tools represents a major and popular tool which enables strong contacts in crystals to be highlighted (Spackman & McKinnon, 2002 ▸). Fingerprint plots can be made with the software *CrystalExplorer* (McKinnon *et al.*, 2004 ▸) by evaluating the pairs of *d*_i_ and *d*_e_ distances from the Hirshfeld surface to the nearest atom interior/exterior to the surface, respectively. Later the *d*_norm_ quantity was introduced by McKinnon *et al.* (2007 ▸) to identify short contacts, *i.e.* those which are shorter than the sum of van der Waals radii of the two atoms interacting. Molecular shape is important in both crystallization and supramolecular assembly and its description using spherical harmonics can be used to classify molecules and their crystal packing (Spackman *et al.*, 2016 ▸).

This Hirshfeld surface analysis can be used in combination with the computation of the different contact enrichment ratios, described by Jelsch *et al.* (2014 ▸, 2015 ▸), to give a statistical picture of the intermolecular interactions in one or a series of crystal packings. The enrichment ratio is a descriptor allowing the analysis in an exhaustive way of the atom–atom contacts in molecular crystals. Thus, it can be used as an indicator of the likelihood of chemical species to form intermolecular interactions with themselves and other species. It is a tool helpful to understand the most important forces in intermolecular interactions, as it provides key information on the distribution of close contacts.

In a first study (Jelsch *et al.*, 2014 ▸), several clear trends were found for contacts in crystals made of organic molecules containing a limited number of chemical species, namely CH, CHO, CHN, CHS and CHF. For instance, in aromatic heterocycles, C⋯C contacts were found to be sometimes highly enriched. Concerning CHO compounds, the different types of O and H atoms were not distinguished and O⋯H contacts were found to be enriched while O⋯O interactions were disfavored.

A second study (Jelsch *et al.*, 2015 ▸) was focused on contact propensities in halogenated hydrocarbons. The halogen⋯halogen contacts and the so-called halogen bonding (Politzer *et al.*, 2007 ▸; Wilcken *et al.*, 2013 ▸), where a halogen makes short interactions with O, N or a π interaction with C, were found to be generally disfavored, except when H is scarce on the molecular surface. While hydrogen is a good partner for halogens, as they are considered as weak hydrogen-bond acceptors (Brammer *et al.*, 2001 ▸), the level of H-atom electropositivity does matter. It was found in oxygenated/halogenated compounds that organic halogen atoms like to make hydrophobic contacts with Hc (hydrogen bound to carbon) while oxygen is rather involved in hydrophilic type contacts, such as O⋯Ho hydrogen-bond interactions.

O atoms can belong to several chemical groups which result in relatively different chemical and electrostatic properties. In the current study, several families of oxygenated hydro­carbon molecules were retrieved from the CSD, Version 5.35, in order to investigate the preferential partners of O atoms in crystal contacts. The propensity of different types of intermolecular contacts to form were analyzed. The enrichment ratios were probed for the intermolecular contacts, especially those involving O atoms. The preferred contact partners of O atoms were compared and the behavior discrepancies between the different oxygen chemical groups are highlighted.

## 2. Materials and methods   

### 2.1. Hirshfeld surface and contacts analysis   

**Figure 1:**
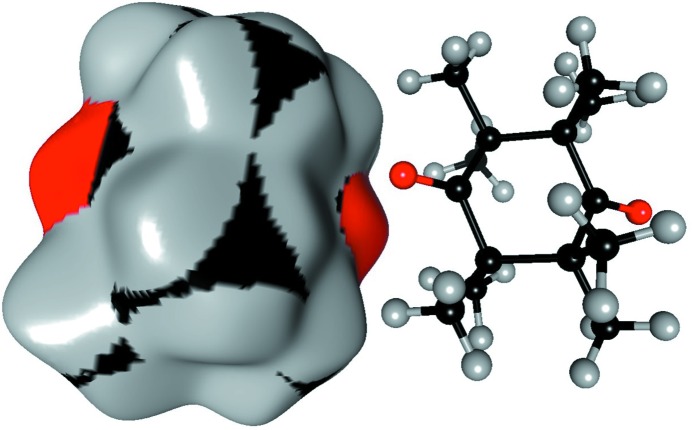
Example of Hirshfeld surface around a molecule in the crystal. The surface is colored according to the type of atom contributing most to the spherical electron density: carbon, hydrogen and oxygen are in black, grey and red, respectively. A symmetry-related molecule which is involved in some O⋯O interaction is shown next to the surface. The ketone compound is octamethyl-1,4-cyclohexanedione of refcode OMCHDO (Hoffmann & Hursthouse, 1976 ▸).

The Hirshfeld surface partitions the space based on the electron density of a molecule (Hirshfeld, 1977 ▸). A molecular weight function which takes values in the [0,1] interval is defined as follows
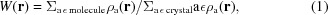
where ρ_a_(*r*) are atomic electron density functions centered on the nuclei of the atoms. In the numerator and denominator, the electron density is summed on the reference molecule and on the crystal, respectively. The appropriate sums of the electron density of the atoms belong to the molecule and the surrounding crystal, respectively. The region of space where *W*(**r**) = ½ is called the Hirshfeld surface which partitions the molecule from its neighbors in the crystalline environment. Inside the Hirshfeld surface, the contribution from the reference molecule dominates over the symmetry-related molecules in the crystal (Spackman & Byrom, 1997 ▸; McKinnon *et al.*, 2004 ▸). The computation of the Hirshfeld surface and of enrichment ratios calculation were carried out with the software *MoProViewer* (Guillot *et al.*, 2014 ▸) and an example is illustrated in Fig. 1 ▸.

The software computes at first two three-dimensional grids containing the electron densities ρ_M_ and ρ_C_ generated respectively by the reference molecule and by its environment. A fast calculation of the spherical atom electron density is proposed although a multipolar atom modelling is also possible. Then, a three-dimensional grid containing the values *W* = ρ_M_/(ρ_M_ + ρ_C_) is generated and the Hirshfeld surface can be displayed by choosing an iso-contour level of ½. The positions of the H atoms were adjusted according to the average bond distances derived from neutron diffraction experiments (Allen *et al.*, 2004 ▸).

In addition, the atomic number *Z* of the atom contributing most to the local electron density is stored in two grids corresponding to the reference and the surrounding symmetry-related molecules. Therefore, the displayed Hirshfeld surface can be colored according to the values *Z*_i_ and *Z*_o_ of the inner or outer atom contributing most to the electron density. Different types of O and H atoms can optionally be distinguished, such as H bound to carbon or oxygen.

The Hirshfeld analysis tool retrieves the following information:

the chemical content of the surface: *S* *x*;

the surface involved in each type of contact: *C*_*XY*_;

the random equiprobable contact surfaces: *R*_*XY*_;

the enrichment ratios: *E*_*XY*_ = *E*(*X*,*Y*) = *C*_*XY*_/*R*_*XY*_.

The enrichment ratio *E*_*XY*_, a descriptor directly derived from the Hirshfeld surface analysis is an indicator of the propensity for two chemical species (*X* and *Y*) to be in contact in crystal packings. It is defined as the ratio between the proportion of actual contacts *C*_*XY*_ in the crystal and the theoretical proportion of random equiprobable contacts *R*_*XY*_. The random contact *R*_*XY*_ values are calculated from the corresponding *S*_*X*_ and *S*_*Y*_ proportions by using the probability products. The value of *E*_*XY*_ is expected to be generally larger than unity for pairs of elements with a high propensity to form contacts in crystals, while pairs that tend to avoid contacts are associated with *E*_*XY*_ values lower than unity. For a crystal made of a unique compound, the chemical contents of the inner and outer surface are, by nature, very similar; therefore, the reciprocal contacts *X*⋯*Y* and *Y*⋯*X* can conveniently be merged.

**Figure 2:**
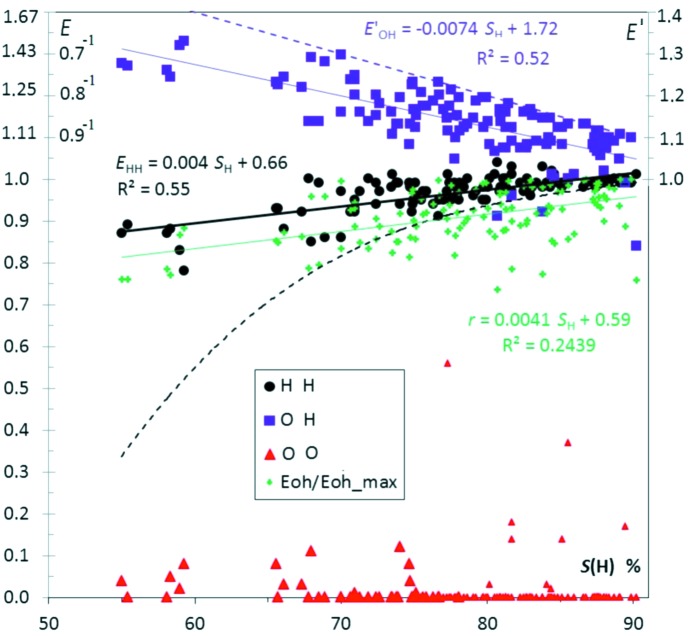
Contact enrichment ratios in crystals of ketones as a function of *S*_H_, the hydrogen proportion on the Hirshfeld surface. In all the graphs, for a more compact representation, the scale for *E* values larger than unity has been modified. For a better graphical representation, *E* values > 1 are replaced by *E*′ = 2 − 1/*E*, in order to obtain *E*′ values in the interval [0., 2.]. In this way, two inverse enrichment ratios are located at the same distance on each side of the line *E* = 1. The *E*′ and *E* scale are both represented in this first graph. Points corresponding to small denominators *R*_*xy*_ < 2% are shown in a smaller size, as they correspond to ratios of small actual and random surfaces. The purple dotted line represents 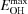, the mathematically highest possible enrichment 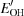 value if the whole oxygen atom surface is involved in O⋯H contacts. The maximum contact surface is 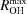 = *S*_O_, therefore the upper limit is 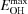 = *S*_O_/(*S*_O_ × *S*_H_) = 1/ *S*_H_. The black dotted line represents the lowest possible *E*_HH_ value occurring when all C and O atoms interact with H: 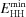 = (2 × *S*_H_ − 1)/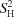. The linear fits on contact enrichments *versus* surface content are performed for all figures in *EXCEL* using the *E*′ values.

In order to have more compact (*S*_*X*_,*E*_*YZ*_) scatterplots, the ordinate values were rendered less dispersed by replacing the *E* ratios by *E*′, where *E*′ = *E* if *E* < 1 and *E*′ = 2 − 1/*E* when *E* is larger than unity (see Fig. 2 ▸). The resulting *E*′ descriptors take values in the [0, 2[ interval. The linear fit lines, multiple regressions and correlation coefficients are computed on the *E*′ values throughout this study. The ‘average *E* values’ in this study were obtained by averaging *E*′ values and the resulting 〈*E*′〉 were back-transformed in 〈*E*〉 values in the following way
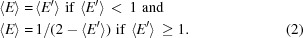
This way, *E* < 1 values are averaged arithmetically, while *E* > 1 values are averaged harmonically.

### 2.2. Selection of molecules   

The crystal structures for several families of oxygenated hydrocarbon molecules were retrieved from the Cambridge Structural Database (CSD; Allen, 2002 ▸) searches. The families of structures were retrieved from the CSD using combinations of criteria, such as notably the chemical species present and the presence of the desired oxygen chemical function(s). To limit the number of entries retrieved, the absence of some other frequent oxygen chemical functions was also required. An example of protocol to search molecules in the CSD is given in the supporting information for the chloro-ether compounds. Molecules with H atoms missing or with disorder were not retained. In general, only structures with a single molecule in the asymmetric unit and no solvent were kept. A number of molecules with a hydroxyl substituent were discarded as the crystal structure showed a wrong orientation of the H atom, leading to short O—H⋯H—O contacts. These structures were detected by unusually high *E*_OO_ enrichment ratios and checked on a computer graphical display. The list of used and discarded compounds is given in the supporting information. In the selected chlorinated molecules, some parts can be aromatic rings, while the ethers, esters, alcohols and ketone were aliphatic hydrocarbons.

### 2.3. Monohydrate and dimers   

It is well known that water molecules play an important role in the structure of bioorganic and organic systems. The presence of water molecules in structures not only mediates biological processes and molecular recognition, but can also control and govern packing formation. In order to gain key information about the interactions of water molecules in organic crystal structures, families of alcohol monohydrate and alcohol/ketone monohydrate molecules were retrieved from the CSD.

Dimers and multimers in the asymmetric unit (*Z*′ > 1) were generally rare in the families of molecules retrieved. However, hydrocarbons substituted simultaneously with hydroxyl and ketone O atoms turned out to have more often two independent molecules in the asymmetric unit. To analyse a dimer, the Hirshfeld surface and contacts were computed on an ensemble of two independent molecules which are not in mutual contact in the crystal. Similarly for monohydrate crystal structures, a water molecule not interacting with the host organic molecule was used.

## 3. Results   

### 3.1. Oxygenated hydrocarbons without the OH group   

#### 3.1.1. Ketones   

Ketones contain Hc hydrogen donor atoms along with oxygen acceptors. Taylor & Kennard (1982 ▸) observed that H atoms that are covalently bonded to carbon have a significant tendency to form short intermolecular contacts to O atoms rather than to H atoms. C—H⋯O interactions are considered as weak hydrogen bonds or van der Waals interactions, depending on the geometry (Steiner & Desiraju, 1998 ▸).

Molecules such as ketones do not have a H atom bound to oxygen; therefore, these compounds are devoid of a strong hydrogen-bond donor and only weak C—H⋯O hydrogen bonds can be formed.

Fig. 2 ▸ shows several enrichment values for aliphatic hydrocarbons substituted with ketone groups as a function of *S*_H_, the proportion of hydrogen content on the surface. In the scatterplot, O⋯H hydrogen bonds appear generally enriched, while H⋯H contacts are slightly impoverished. On the other hand O⋯O contacts are absent or very scarce.

When a chemical species is preponderant on the molecular surface, some limitations apply on the related enrichment values. For instance when *S*_H_ tends to 100%, for mathematical reasons the *E*_HH_ value tends to unity (*C*_HH_ > 2 × *S*_H_ − 1, therefore *E*_HH_ > [2 × *S*_H_ − 1]/*S*_H_ *^2^*). For *S*_H_ = 70%, the lower limit of *E*_HH_ is still 0.82. This effect is visible in the Fig. 2 ▸ displaying *E*_HH_ values as a function of *S*_H_ for ketone compounds.

In addition, when the chemical composition on the surface is not far from 100% of hydrogen, the maximal value of *C*_OH_ is 2 × *S*_O_ (the factor 2 originates from the reciprocal contacts O⋯H and H⋯O which are counted together). The value of *R*_OH_ is 2 *S*_O_ × *S*_H_ and as a consequence *E*_OH_ = *C*_OH_/*R*_OH_ <1/*S*_H_. Therefore, a negative slope of *E*_OH_ for large values of *S*_H_ (Fig. 2 ▸) cannot be interpreted solely as a diminishing likelihood of O⋯H contacts to form. The theoretical maximal value of *E*_OH_ is illustrated in Fig. 2 ▸ and shows that several compounds, with hydrogen proportion *S*_H_ between 70 and 90%, have *E*_OH_ values close to the maximal limit.

The fitted lines (*E*′ *versus S*_H_ in Fig. 2 ▸) and their slopes indicate the average tendencies of the contact enrichments with chemical composition (Jelsch *et al.*, 2014 ▸). The enrichment of O⋯H contacts is highest for ketones not so rich in hydrogen. The comparison of the *E*_OH_ and *E*_HH_ fitted lines with the theoretical max(*E*_OH_) and min(*E*_HH_) values in Fig. 2 ▸ suggests, however, that part of the (*E*,*S*_H_) trends originate from the mathematical limitations mentioned. Another indicator, the ratio *r* = *E*_OH_/*E*_OH_max_, is also plotted in Fig. 2 ▸; it indicates that the O⋯H enrichments reach, on average, 91% of their maximal theoretical value, with a sample standard deviation (s.s.d.) of 7%. In other words, 91 ± 7% of the oxygen Hirshfeld surface interacts with hydrogen, and the proportion stays within the limits 73 to 99.7%.

The case of ketone molecules was further investigated by multiple linear regression to see if the 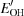 values can be better fitted than in the simple regression line in Fig. 2 ▸ using several chemical proportions as well as the molecular weight *W*. The single linear regressions of 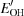 show similar correlations of 45% fittings for *S*_O_ and S_C_ proportions, while the *E*_OH_ values show a much higher dependency on the hydrogen content *S*_H_ with *r* ^2^ = 52% (Table 1 ▸). The application of multiple linear regression using [*S*_H_,*S*_O_] and [*S*_H_,*S*_O_,*W*] results in similar correlation coefficients found in the [0.523, 0.526] range. The use of up to three variables did not improve the fitting, suggesting that enrichments of the O⋯H contacts depend mostly on the hydrogen content. The molecular weight was not found to have an influence on the propensity of ketones to form C—H⋯O hydrogen bonds in the crystal.

#### 3.1.2. Ethers   

**Figure 3:**
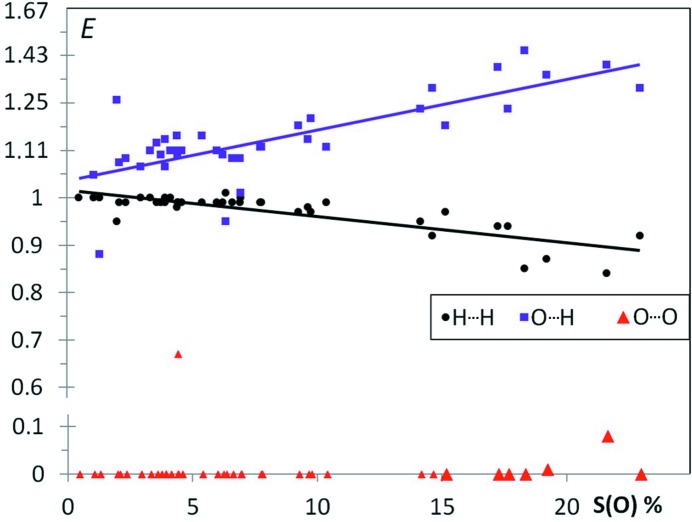
Contact enrichment ratios in crystals of ethers as a function of *S*_O_.

Fig. 3 ▸ shows the (*S*_O_, *E*) scatterplot for 40 aliphatic ether compounds. The O⋯H enrichment values for ethers 〈*E*〉 = 1.14 (1) have, on average, very similar values compared with ketones 〈*E*〉 = 1.16 (1). Fig. S1 shows comparable enrichments *E*_OH_ for ethers and ketones on the same graph as a function of *S*_H_, the hydrogen content on the surface. Despite the fact that the ether function is less hydrophilic and forms weaker hydrogen bonds than the ketone group, O⋯O contacts are similarly avoided in both classes of compounds, due to electrostatic repulsion.

#### 3.1.3. Nitro-ethers   

**Figure 4:**
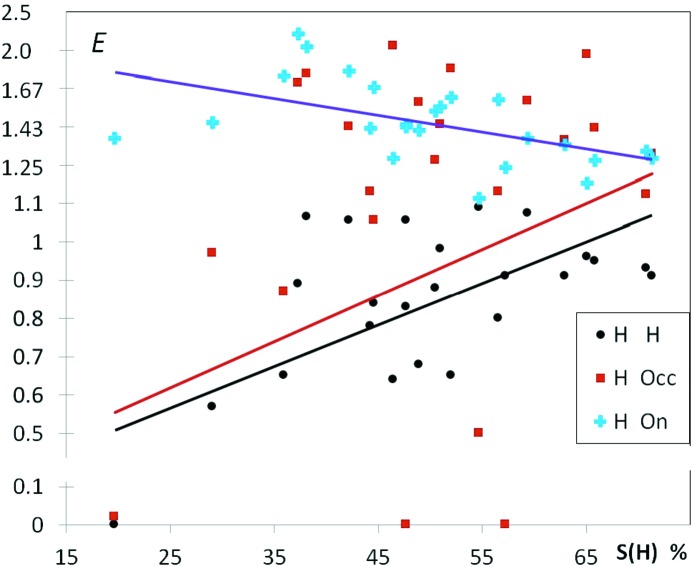
Contact enrichment ratios in crystals of hydrocarbons substituted with both nitro and ether groups. Occ and On refer to the ether and nitro oxygen atom types, respectively.

Hydrocarbon compounds (aliphatic and aromatic) substituted with both a nitro and an ether group are analysed in Fig. 4 ▸. The O⋯H—C interactions have a slightly higher propensity to occur for nitro groups than for ethers. This result suggests that, in an environment devoid of hydrophilic H atoms, the nitro O atoms exhibit a slightly more pronounced hydrogen-bond acceptor character than the ether oxygen. The presence of two close O atoms presumably generates a slightly more electronegative potential around a nitro group compared with the ester O atom which makes it more attractive to very weakly charged H—C atoms. The donor and acceptor strengths based on mean distances in C—H⋯O hydrogen bonds were quantified by Steiner (1998 ▸) in a crystallographic database study; the nitro and ether groups were included in the investigation. The Hc⋯O distances were not systematically shorter for one of the two moieties, rather the relative distances were strongly dependent on the nature of the C—H donor. For strong donors such as water or NH_3_^+^, the nitro group was ranked as a weaker acceptor than the ether.

#### 3.1.4. Esters   

**Figure 5:**
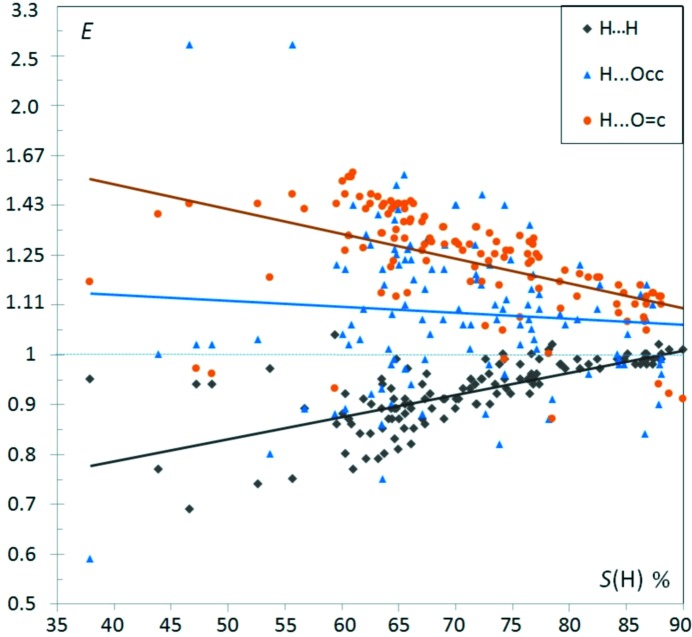
Contact enrichment ratios in crystals of esters as a function of *S*_H_. Occ and O=c refer to the ether and carbonyl oxygen atom types, respectively.

Molecules composed of aliphatic hydrocarbon fragments and ester groups were retrieved from the CSD. These compounds possess two types of O atoms: the O=c oxygen atom with one double bond and Occ forming two single bonds with carbon. As illustrated by the Fig. 5 ▸, the O=c⋯H and Occ⋯H contacts are generally both favored. The relative positions of the fitted curves of enrichment ratios as well as the average values of 〈*E*(O=c,H)〉 = 1.23 (1) and 〈*E*(Occ,H)〉 = 1.07 (1) sustain that the O=c oxygen atom is more electronegative and is a stronger hydrogen bond acceptor than the Occ atom. This result is in agreement with the Steiner (1998 ▸) structural study of several C—H⋯O-type hydrogen bonds which classifies carbonyl as a stronger acceptor than ether. Average H⋯O distances were found to be smaller for O=c compared with Occ for all types of weak and strong H donors. In esters, like in the three other families of compounds devoid of a hydroxyl group, the H⋯H contacts are generally slightly under-represented, as already observed for both aliphatic and aromatic oxygenated compounds (Jelsch *et al.*, 2014 ▸).

### 3.2. Alcohols and phenols   

**Figure 6:**
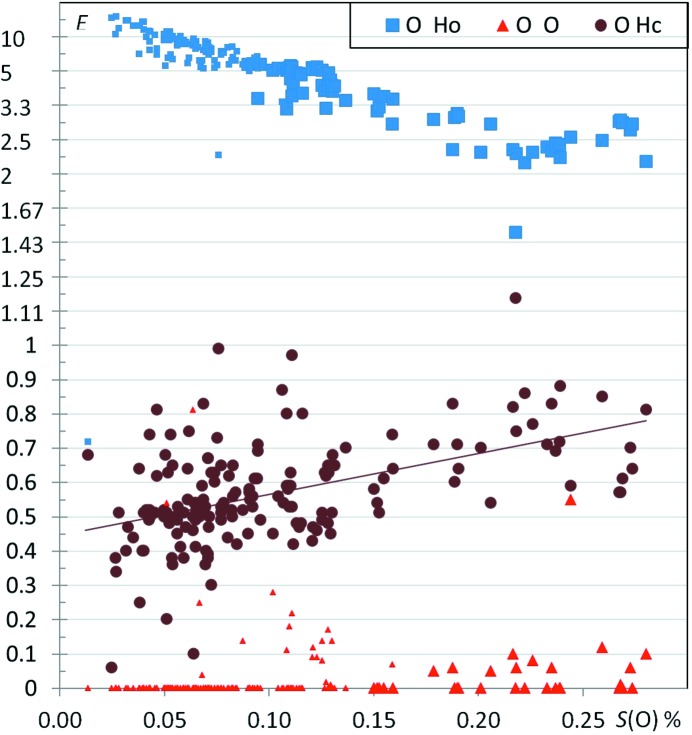
Contact enrichment ratios in crystals of alcohols as a function of *S*_O_. Ho and Hc refer to the hydrogen bound to oxygen and carbon atoms, respectively. Small symbols correspond to cases where the equiprobable contacts *Rxy* are smaller than 2%.

**Figure 7:**
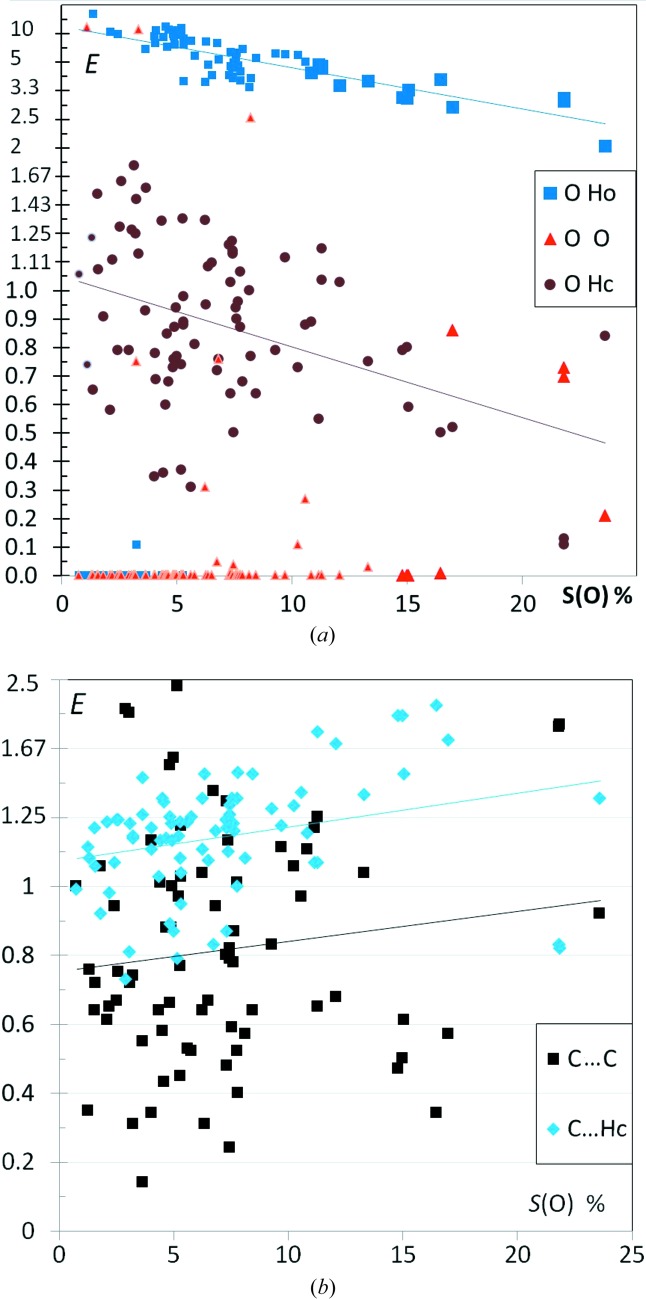
(*a*) Enrichment of O⋯O, O⋯Hc and O⋯Ho contacts in phenols. Small symbols correspond to cases where the equiprobable contacts *Rxy* are smaller than 2%. (*b*) Enrichment ratios for the hydrophobic contacts types C⋯Hc and C⋯C.

Aliphatic hydrocarbons with C*sp* ^3^—OH hydroxyl substituents (referred as alcohols) were retrieved from the CSD and are analyzed in Fig. 6 ▸. Hydrocarbons substituted with phenol O atoms were also searched for comparison (Fig. 7 ▸). These two hydroxyl functions are considered as strong hydrogen-bond acceptors and donors, but are characterized by different p*K_a_* values, with phenols losing their proton when the pH is above 10. Gilli *et al.* (2009 ▸) have investigated the relationships between hydrogen-bond strengths and acid–base molecular properties (p*K_a_* rule). From an electron density point of view, the two electron lone pairs of the O atom appear to be in a closer configuration in phenols than in alcohols, due to resonance effects with the neighbor aromatic ring (Domagała *et al.*, 2012 ▸). As a result, the hydrogen bond patterns of the two oxygen acceptors display different preferential directions for the H⋯O interactions in the crystal structures found in the CSD (Ahmed *et al.*, 2013 ▸).

**Figure 8:**
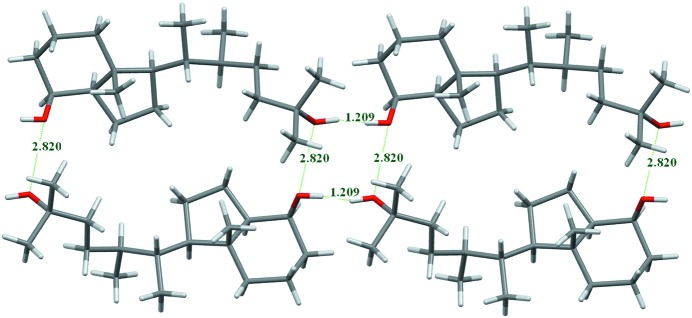
An example of an alcohol for which the crystal structure has some errors as detected by high *E*_OO_ values. A tetramer of the published structure with refcode TEZQIA in the CSD is shown. The two hydroxyl hydrogen atoms on atoms O1 and O2 are obviously wrongly oriented as the distance between them is only 1.21 Å. In addition, there are non-favorable O⋯O contacts at a distance of 2.82 Å instead of an O—H⋯O hydrogen bond.

The electronegative O atoms avoid generally interacting with themselves as this is electrostatically unfavorable (Figs. 6 ▸ and 7 ▸ *a*). Some molecules however showed strong *E*(O,O) enrichments larger than 2.5. Oxygen–oxygen contacts in crystals have been discussed from an energetic point of view (Gavezzotti, 2010 ▸); they do not occur alone but they are generally secondary to stronger interactions. Careful inspection of these structures on computer graphics reveals that some Ho atoms are incorrectly placed, as hydroxyl groups present a rotational degree of freedom. For instance, the molecule with the refcode TEZQIA in the CSD (C_19_H_36_O_2_; Flores *et al.*, 2012 ▸) has two hydroxyl groups forming an O—H⋯H—O interaction resulting in a too short Ho⋯Ho distance of 1.21 Å (Fig. 8 ▸).

Among the alcohol compounds, as many as 13 molecules with *E*_OO_ ranging between 0.6 and 20 had to be discarded due to obvious misplacement of Ho atoms in view of the geometric configuration of the hydroxyl groups in the crystal. The molecules with low O⋯Ho hydrogen-bond content were also inspected and six crystal structures with *E*(O,Ho) < 1.7 were omitted due to incorrect hydroxyl group positioning. The 144 retained molecules and the discarded ones are listed in the supporting information. In a large majority of the remaining alcohol compounds, there are actually no O⋯O contacts.

Among phenols, an outlier (refcode AKUSII; Stanciu *et al.*, 2003 ▸) with a large *E*_OO_ = 12.8 shows an oxygen⋯oxygen contact through an inversion center, but at a relatively long distance of 3.91 Å. According to the authors, the large terphenyl substituents prevent hydrogen-bonded association of the phenols and this compound has indeed an *E*(O,Ho) value of zero. The compound with refcode KAZHAU (C_22_H_16_O) from the same reference (Stanciu *et al.*, 2003 ▸) is also an outlier with *E*_OO_ = 11.8 and the interatomic O⋯O distance is 2.76 Å, which is too short for this type of contact (Fig. S2). The intermolecular interaction occurs through an inversion center and the correct structure for this compound has presumably a Ho hydrogen atom with two positions at half occupancy as proposed in Fig. S2. In the alternative position, an O—Ho⋯O hydrogen bond is formed.

The enrichments *E*(O,Ho) are almost always larger than unity and the average trends show a negative slope with the proportion of oxygen on the molecular surface for both alcohols and phenols (Figs. 6 ▸ and 7 ▸ *a*). This means that compounds with a small number of O atoms have most of the oxygen and polar hydrogen Ho atoms involved in O⋯Ho strong hydrogen bonds. For compounds with several O atoms, it is, from a geometric and crystal packing point of view, more difficult to have all of them interacting simultaneously with a Ho atom.

A point at position *S*_O_ = 7.6 and *E*(O,Ho) = 2.25 in Fig. 6 ▸ which appears as distant from the average trend in the scatterplot is an alcohol with only moderately enriched O⋯Ho contacts. The corresponding refcode POJYIX molecule (C_19_H_30_O_4_; Anderson *et al.*, 2007 ▸, Fig. S3) is peculiar as it has three intramolecular hydrogen bonds which link four hydroxyl groups which are nearly aligned on one side of the molecule.

For phenols, there are some 15 outlier compounds with a low proportion of oxygen on the surface which do not form any O—H⋯O strong hydrogen bond [*E*(O,Ho) = 0]. This occurs for example for the compound with the refcode AKUSEE (C_36_H_50_O; Stanciu *et al.*, 2003 ▸; Fig. S4) which is a large molecule where the hydroxyl group is surrounded by bulky phenyl groups and, presumably for steric reasons, forms weak C—H⋯O interactions instead of O—H⋯O hydrogen-bonds. The phenol compound of refcode JABVUD (C_36_H_38_O_4_; Goldmann *et al.*, 1998 ▸; Fig. S5) is an outlier for another reason, the four hydroxyl groups of the molecule form a network of intramolecular O—H⋯O hydrogen bonds while the intermolecular contacts are of the type C—H⋯O [*E*(O,Hc) = 1.34]. The compound of refcode TUJYAZ (C_29_H_20_O; Debeaux *et al.*, 2009 ▸) has a unique hydroxyl group which forms an intramolecular O—H⋯π weak hydrogen bond (Levitt & Perutz, 1988 ▸) with an aromatic cycle (Fig. S6). All other phenol outliers follow one of these three schemes. In the phenol compounds, when no or not all strong O—H⋯O hydrogen bonds occur, the weaker Hc⋯O interactions are instead enriched (Fig. 7 ▸ *a*).

The enhancement ratios of Ho⋯O interactions in alcohols are always significantly larger than the *E* ratios of C—H⋯O weak hydrogen bonds, suggesting that the formation of strong Ho⋯O hydrogen bonds is a main driving force in the crystal packing formation for this type of molecule. The Ho⋯O strong hydrogen bonds are considerably more enriched in alcohols [*E*(Ho,O) is often larger than 2.5] compared with the weaker Hc⋯O interactions in ketones, which have a strong oxygen acceptor but no strong hydrogen-bond donor.

In the case of alcohols, the O⋯Hc interactions are nearly always disfavored with *E*(O,Hc) < 1. The average value of *E*(O,Hc) ratios tends to increase from 0.5 to 0.8 when the proportion of oxygen on the surface augments. For molecules with a high proportion of O atoms, the increase of *E*(O,Hc) ratios together with the lower values of *E*(O,Ho) can be understood as the geometric impossibility to involve all the O atoms in a strong O⋯Ho hydrogen bond, therefore the remaining O atoms tend to form a weak O⋯Hc hydrogen bond.

If Ho and Hc hydrogen atoms were not distinguished, the limiting law *E*_OH_ < 1/*S*_H_, which is illustrated for ketones in Fig. 2 ▸, would apply. As a consequence, for compounds rich in hydrogen, which is correlated with low content in oxygen, the high *E*(O,Ho) values imply correlatively decreased *E*(O,Hc) ratios, which can be graphically observed in Fig. 6 ▸.

The case of phenols shows several differences compared with alcohols with larger *E*(O,Hc) values for molecules poor in oxygen. A mathematical explanation comes from the upper limit of *E*(O,Hc) which is *S*_O_/[*S*_O_ × *S*_Hc_] = 1/*S*_Hc_. In the case of phenols with significant carbon content on the surface, *S*_Hc_ takes smaller values than in aliphatic alcohols; as a result the upper limit of *E*(O,Hc) is larger for phenols. Another difference with alcohols is the negative slope in phenols for *E*(O,Hc) as a function of *S*_O_. The diminishing *E*(O,Hc) could be related to the fact that the hydrophobic Hc atoms are also attracted by the large carbon surfaces found in aromatic ring systems of phenols. Fig. 7 ▸(*b*) shows that C⋯Hc contacts are generally enriched and the trend increases with *S*_O_. It has to be reminded here that there are some dependencies between the surface atom type components, the proportions *S*(Ho) and *S*(C) are 96 and 34% correlated with *S*(O), respectively, while *S*(Hc) is anti-correlated (Fig. S7). In a previous study (Jelsch *et al.*, 2014 ▸), it was found that purely aromatic oxygenated compounds (CHO) had generally very high *E*_CC_ values due to extensive π⋯π stacking, while C⋯Hc interactions were disfavored. Conversely, the phenol hydrocarbon compounds studied here contain generally both an aromatic and an aliphatic part and follow a different behavior. They show C⋯C contacts which can be sometimes very enriched but are more often rather impoverished. On the other hand, Hc⋯C contacts are generally enriched and preferred to C⋯C contacts. Phenol hydrocarbons also show a higher propensity for Hc⋯O contacts [〈*E*〉 = 0.88 (3)] compared with alcohols [〈*E*〉 = 0.56 (1)]. One contributing factor is that the H atoms on aromatic groups, due to the withdrawing effect of the aromatic π system, are slightly more electropositive than on aliphatic parts. The charges of H atoms in phenylalanine are, for instance, 0.115 e on the aromatic ring and 0.09 e on the aliphatic part in the CHARMM36 force field (Best *et al.*, 2012 ▸).

### 3.3. Alcohol–phenol compounds   

**Figure 9:**
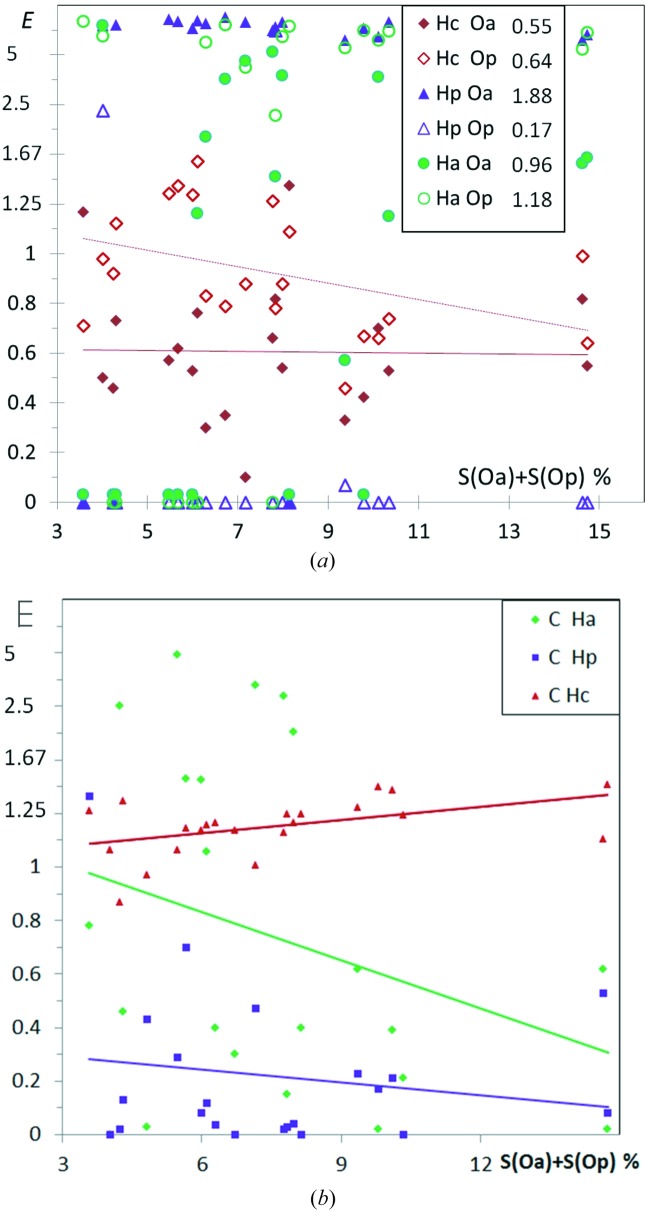
(*a*) Enrichment of six different types of O⋯H contacts in alcohol–phenol hydrocarbons. There are three types of hydrogen atoms, Hc, Ha and Hp and two types of oxygen atoms Oa and Op. The suffixes ‘a’ and ‘p’ refer to alcohols and phenols, respectively. The average *E*′ values are given in the legend. (*b*) Enrichment of different C⋯H contacts.

21 hydrocarbons substituted with both alcohol and phenol O atoms have also been retrieved from the CSD and analyzed (Fig. 9 ▸ *a*). There are six different chemical types which are considered, and the number of H⋯O interaction types reaches six. Therefore, in such a situation with many atom type subdivisions, some favorable interactions can happen to be incidentally absent. The most enhanced hydrogen bonds appear to be cross contacts between alcohols (‘a’ suffix) and phenols (‘p’ suffix), namely Hp⋯Oa followed by Ha⋯Op with average enrichment values of 2.69 (16) and 1.25 (20), respectively. On the other hand, many structures show an absence of some types of O—H⋯O hydrogen bonds. Hydrogen bonding between alcohol moieties shows several enriched *E*(Ha,Oa) values around 2.2 with 0.4 s.s.d. together with several zero *E* values; as a result the average *E* value is at 0.90 (17) slightly lower than unity. Unexpectedly, strong hydrogen bonds within phenols are very under-represented in alcohol/phenol compounds with 〈*E*(Hp,Op)〉 = 0.18 (12), as 18 out of 20 crystal structures display no such contact. The hydrophobic H atoms interact generally slightly more with phenol than with alcohol acceptors, the Hc⋯Oa contacts being clearly disfavored with 〈*E*〉 = 0.61 (6).

**Figure 10:**
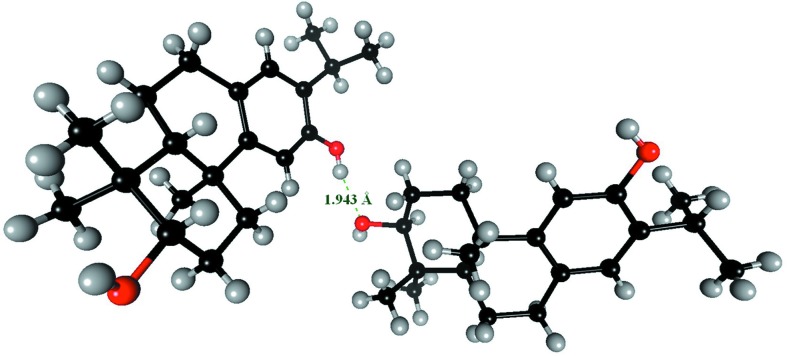
Hinokiol crystal structure (TITHOU compound in CSD; Li *et al.*, 2007 ▸), a typical case of alcohol/phenol hydrocarbon where not all hydrogen-bond acceptors and donors find a partner. The phenol group is a hydrogen-bond donor to the alcohol acceptor (Op—Hp⋯Oa interaction). The phenol oxygen acceptor forms weak hydrogen bonds with Hc atoms, while the Ha atom is involved in an Oa—Ha⋯π interaction with the phenol group (not shown).

The graph of Fig. 9 ▸(*b*) shows the enrichment contact ratios of C⋯H interactions in alcohol + phenol hydrocarbons as a function of oxygen content on the surface. The C atoms display more contact affinity with Hc than with Hp. The C⋯Hp contacts are indeed very impoverished. This is due to the fact that the Hp hydrogen atoms bound to the phenolic oxygen are more electropositive than Hc and for electrostatic reasons Hp atoms are more likely to form contacts with O rather than C atoms. Ha alcohol H atoms are less electropositive than the phenolic ones and the behavior of Ha⋯C contacts is indeed more contrasted with a large range of *E*(Ha,C) values between 0 and 5. In several cases, Ha⋯C contacts can be significantly favored, even surpassing in enrichment the hydrophobic Hc⋯C contacts. A typical case is presented in Fig. 10 ▸ for compound TITHOU (C_20_H_30_O_2_; Li *et al.*, 2007 ▸) which has one phenol and one alcohol group, the crystal packing driving force seems to be the formation of a Hp⋯Oa hydrogen bond while the hydrogen bonding capacity of the Ha and Op charged atoms is sacrificed.

### 3.4. Alcohol/ketone compounds   

**Figure 11:**
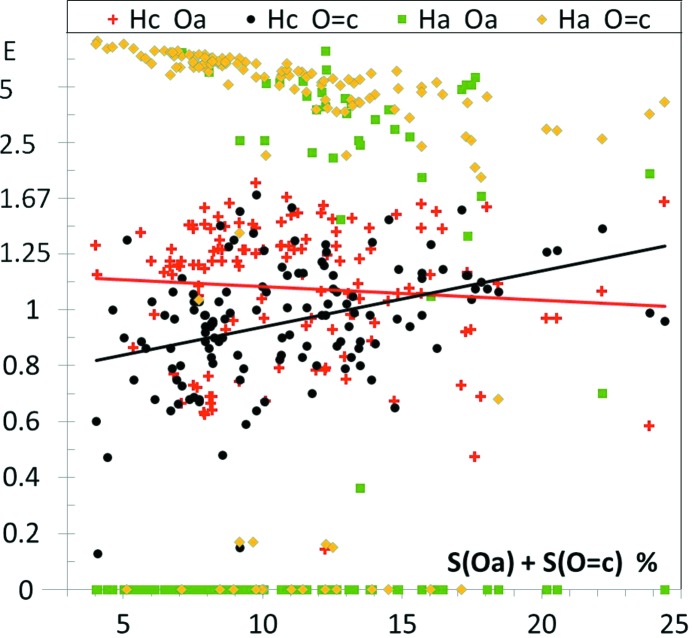
The enrichment of different hydrogen atom types in alcohol–carbonyl.

The crystal contact propensities involving H and O atoms in hydrocarbons substituted with both hydroxyl and ketone functional groups are analyzed in Fig. 11 ▸. These structures have two types of O atoms O=c (ketone) and Oa (alcohol) and two types of H atoms Hc and Ho. The two possible strong hydrogen bonds, Ha⋯O=c and Ha⋯Oa, often show high enrichment values, but due to the large number of atoms types, a significant number of electrostatically favorable contacts are incidentally very impoverished or absent. Globally the Ha⋯O=c hydrogen bonds with ketone oxygen acceptors are more enriched than those involving hydroxyl oxygen acceptors (Ha⋯Oa) which, on the other hand, are more often absent in the crystal packing. These tendencies confirm that the ketone group is a stronger hydrogen bond acceptor than the hydroxyl moiety. For com­pounds poor in hydrogen, the hydrogen-bonding involving ketone oxygen acceptors Ha⋯O=c are generally extremely enriched to the detriment of the alcohol oxygen acceptors which then often form no strong Ha⋯Oa hydrogen bond.

The Hc hydrogen atoms constitute a larger part of the surface in these molecules; as a result, both types of weak hydrogen bonds Hc⋯Oa and Hc⋯O=c do occur in all the crystal packings (*E* > 0.1). These interactions show enrichment values generally around 1.0 ± 0.4. The weak hydrogen bonds involving alcohols seem, however, slightly more favored than those with ketone. This is directly related to the opposite tendency found for strong hydrogen bonds with a hydroxyl donor.

No strong trends as a function of chemical composition on the molecular surface were found for the different H⋯O interactions. The Hc⋯O=c interactions tend, however, to have increasing likelihood to occur for molecules with high oxygen content.

### 3.5. Molecular dimers in the asymmetric unit   

Among the alcohol–ketone compounds retrieved from the CSD, a total of 20 crystal structures turned out to have *Z*′ = 2, *i.e.* two independent molecules in the asymmetric unit (three molecular structures with wrongly oriented hydroxyl groups were discarded). This constitutes a significant proportion as 133 molecular structures were retrieved with *Z*′ = 1; in addition, two molecules were found to have *Z*′ = 4. Generally, dimers were found to be much rarer in the different families of retrieved oxygenated hydrocarbons. Alcohols are the other notable exception, where 180 monomers in the asymmetric unit were analyzed, but 31 dimers and 12 multimers were also found.

**Figure 12:**
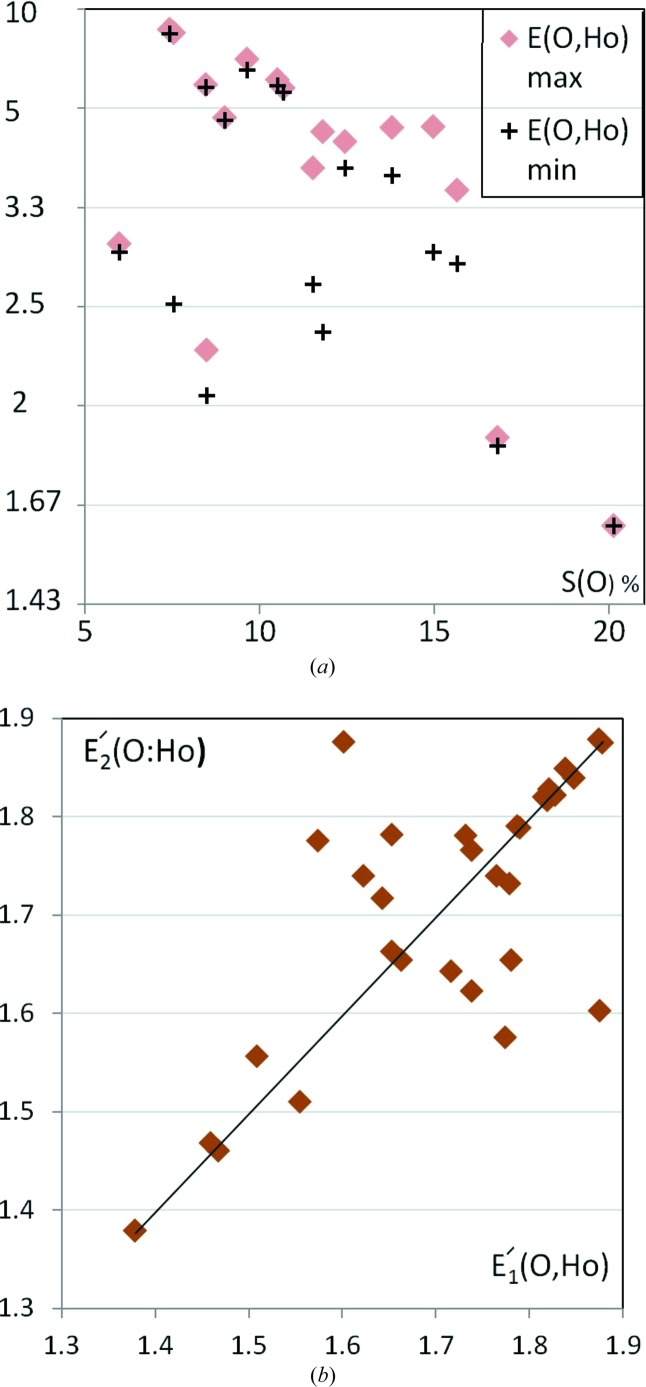
Enrichment of O⋯Ho contacts in hydroxyl–carbonyl hydrocarbons containing a dimer in the asymmetric unit. (*a*) The *E*(O,Ho) values are represented for each independent molecule as a function of oxygen content on the surface. (*b*) The two *E*′(O,Ho) values are represented as a function of each other. The (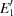, 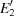) and (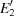, 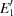) pairs are both represented in order to obtain a symmetric graph which is independent of dimer #1 and #2 attributions.

In this dimers study, the H atoms were differentiated (Hc and Ha) while the two types of O atoms Oa and O=c were not distinguished, in order to limit the number of atom types and to avoid the incidental absences of contacts types in some structures. The enrichment *E*(O,Ha) of the strong hydrogen bonds are specifically analyzed in dimers of ketone/alcohol compounds (Figs. 12 ▸ *a* and *b*). The O⋯Ha contacts are often enriched by a ratio larger than 3.3 and the correlation coefficient between the two sets of *E*(O,Ha) values in the dimers reaches 77% (Table 2 ▸). In most of the crystal structures, the two independent molecules have a very similar amount of strong hydrogen bonding (Fig. 12 ▸ *b*). The correlations between the *E*′ values of dimers for all the interaction types are shown in Table 2 ▸ and the coefficients are in the 0.376–0.825 range. The largest correlations are found for the C⋯O, C⋯Hc and Ho⋯Ho contacts which are above 0.80.

### 3.6. Alcohol monohydrate compounds   

The first attempts to crystallize an organic molecule which is water soluble are often performed in aqueous solution. In a statistical analysis of the CSD, about 8% of the crystal of organic compounds are hydrates according to Görbitz & Hersleth (2000 ▸) on the inclusion of solvent molecules in the structures stored in the CSD. The probability of organic compounds crystallizing as hydrates augments with an increasing number of polar chemical groups in the molecule (Infantes *et al.*, 2003 ▸). In that study, the likelihood of water molecules to be present in the crystal is described for several chemical groups including organic moieties and ions.

**Figure 13:**
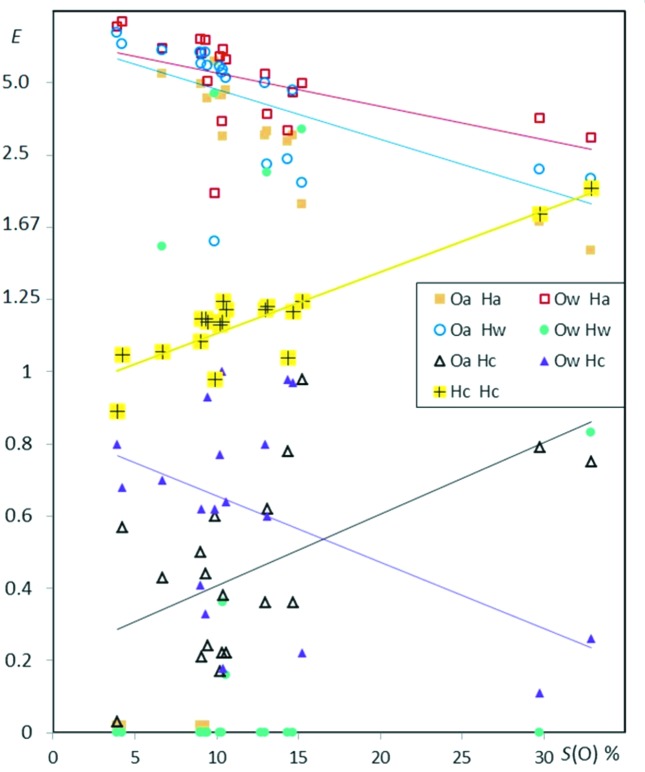
Enrichment of the oxygen⋯hydrogen contacts in crystals of alcohol-monohydrates as a function of total oxygen content on the molecular surface. ‘a’ and ‘w’ suffixes refer to alcohol and water, respectively. The *E* ratios of the Hc⋯Hc hydrophobic interactions are also displayed.

In order to investigate the behavior of water molecules in crystals, 20 aliphatic alcohol-monohydrate crystal structures were retrieved from the CSD (Fig. 13 ▸). One of them was discarded due to wrong hydroxyl hydrogen atom placement, as detected by a high *E*(O,O) value and showing an O—H⋯H—O interaction (Table S1). The different types of O⋯H hydrogen bonds are analyzed; globally most of the strong hydrogen bond types involving water and the hydroxyl groups show enrichment ratios values larger than 2. However, for half of the structures, there is no Hw⋯Ow interaction between water molecules. In a few cases, hydrogen bonding between alcohol groups is also absent. On the other hand, the cross interactions between water and hydroxyl groups are the most enriched. Water molecules are small in size and their placement is more flexible in a crystal packing than that of host organic molecules, therefore they can be driven to be hydrogen-bond partners to the alcohol groups. The interaction of the O atoms with the hydrophobic hydrogen atoms Hc is always impoverished and the *E* values are generally between 0.2 and 1.0 in alcohol monohydrates. The Hc⋯Hc hydrophobic interactions display an enrichment value slightly superior to unity 〈*E*〉 = 1.15 (3).

Fig. S8 shows the different H⋯O contacts for phenol-monohydrate crystals and can be compared with the results for alcohol-monohydrate compounds in Fig. 13 ▸. Despite the small sample sizes, several tendencies are in accordance with the two graphs such as the low occurrence of Ow⋯Hw hydrogen bonds between water molecules, the high enrichment of strong cross hydrogen bonds between the hydroxyl and water moieties and the generally slightly disfavored Hc⋯Ow and Hc⋯O(hydroxyl) weak hydrogen bonds.

In both alcohol-monohydrate and phenol-monohydrate crystals, the presence of water molecules facilitates the formation of hetero-contacts between alcohol/phenol and water moieties. The hydrogen-bond hierarchy in compounds containing both a carboxylic acid/phenol moiety and a chloride anion was studied by the Zaworotko group (Duggirala *et al.*, 2015 ▸). The presence of competing hydrogen-bonding groups such as the water molecule was shown to disrupt the hydrogen-bond network existing in its absence (COOH⋯COOH, COOH⋯Cl^−^, PhOH⋯PhOH, PhOH⋯Cl^−^). The investigation revealed that the inclusion of water molecules in these compounds resulted in the formation of COOH⋯H_2_O and PhOH⋯H_2_O above-molecular hetero-synthons in 83 and 95% of the cases, respectively. These results are in accordance and compatible with those of the current study.

### 3.7. Alcohol–ketone monohydrate compounds   

**Figure 14:**
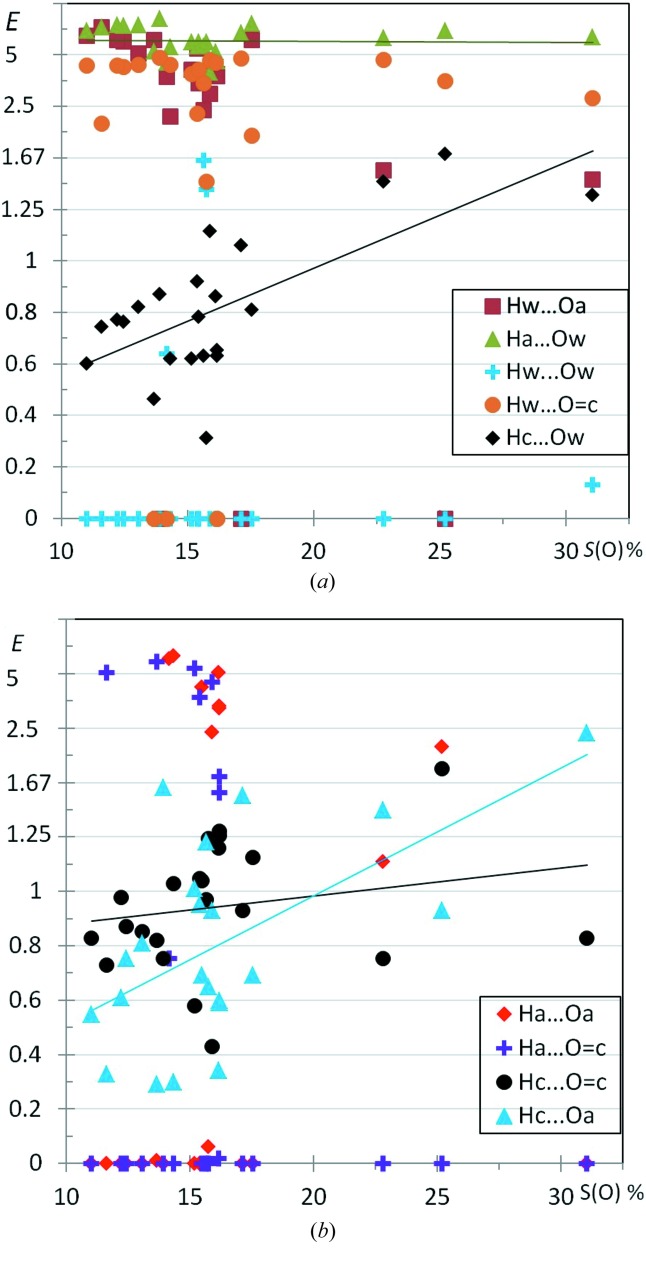
Enrichment ratios of the different hydrogen⋯oxygen interactions in alcohol + ketone monohydrate molecules, as a function of total oxygen content on the molecule surface. Suffixes ‘w’ and ‘a’ refer to water and alcohol, respectively, while O=c refers to ketone oxygen atom. The interactions involving a water atom are in graph (*a*) while the remaining ones are in graph (*b*).

In order to investigate the behavior of water in the presence of two different oxygenated chemical functions, the hydrogen⋯oxygen interactions in a total of 21 monohydrate crystal structures containing alcohol and ketone groups are analyzed in Fig. 14 ▸. The retrieved compound YAHQED (Ito *et al.*, 2000 ▸) was discarded, as it had a high *E*_OO_ ratio and the water H—O—H angle was found to be wrong at 70°.

Three O-atom types are distinguished (ketone, alcohol and water) and two hydrogen types (Ha, alcohol and Hc). The water hydrogen atoms like to interact with ketone and alcohol oxygen acceptors in a similar way and the enrichment ratio is generally larger than 3. The water oxygen acceptors have strongly enriched interactions with the polar H atoms of alcohols.

Although they are also strong hydrogen bonds, interactions between water molecules Hw⋯Ow are generally absent or less enriched, as already observed for alcohol-monohydrates. The other strong hydrogen bonds, not involving water, namely Ha⋯O=c and Ha⋯Oa, have very disparate trends, being either over-represented in the crystal structure or totally absent. The absence of these contacts, notably for molecules poor in oxygen, can be attributed to the small surface proportions involved and the differentiation in many atom types (statistical effect). Another reason, which is stereochemical, is that, with their small size, water molecules can easily occupy interstices between the host organic molecules. The water molecule has an electron density shape close to a sphere and therefore has high orientation flexibility, and it can easily interact with the available hydrogen-bond acceptors and donors in the alcohol and ketone moieties. On average, the Ha⋯Ow hydrogen bonds show the highest enrichments among all contacts (Table 3 ▸), presumably because the water molecule can easily fit in the crystal structure in order to have Ow interacting with the only non-water (non-self) hydrogen-bond donors available which are the alcohols. For the crystallization of relatively large compounds, Görbitz & Hersleth (2000 ▸) recommend actually the use of mixtures containing hydrogen-bond donating and accepting solvents as well as less polar solvents to allow the inclusion of solvent into two or more types of cavities with different properties.

The weak hydrogen bonds of C—H⋯O type show similar trends for all the oxygen atom types. They are generally slightly disfavored when the oxygen content is poor in the molecule, but the *E*(Hc⋯O) values tend to increase with *S*(O).

The conclusions on contacts in monohydrate crystals may be mitigated, as the samples *N* = 19 in alcohol monohydrates and *N* = 21 for alcohol–ketone monohydrates are limited in size. For some contacts, clear tendencies do appear and linear fits can be drawn. Some contacts such as Ow⋯Ha and Oa⋯Hw show similar high *E* values in both Figs. 13 ▸ and 14 ▸, which supports the results, despite the small sample size. Similarly, the Hc⋯Ow contacts are generally disfavored in both figures. Despite the limited sample, some behaviors can clearly be explained. For example, in alcohol–ketone monohydrates, the Ha⋯O=c and Ha⋯Oa contacts are either highly enriched (as attractive contact) or absent (due to the large number of chemical types sub-divisions).

### 3.8. Chloro-ether molecules   

**Figure 15:**
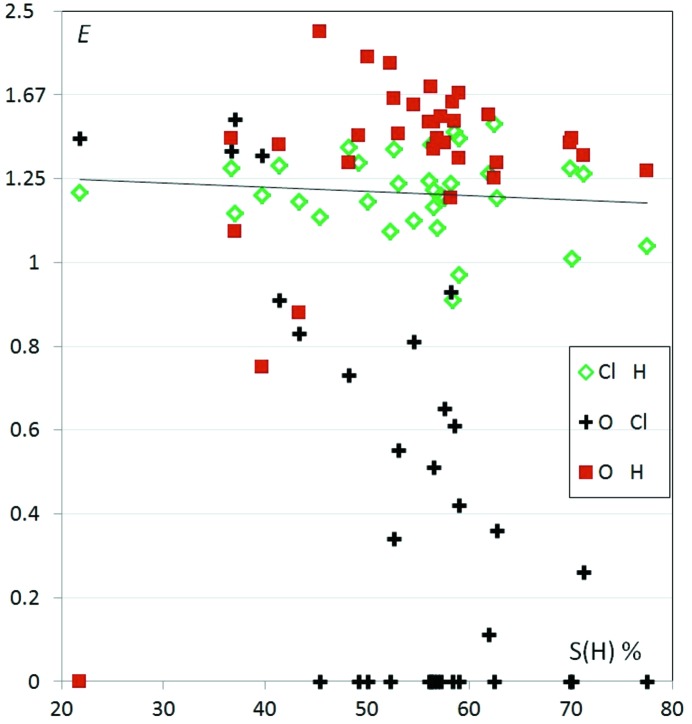
Enrichment of contacts in chlorinated hydrocarbons with ether groups.

In order to analyze the behavior of organic chlorine with some O-atom types like ethers, 33 chlorinated compounds with an ether function were retrieved from the CSD. As can be seen in Fig. 15 ▸, both ether and chlorine moieties like to interact with hydrogen Hc. Organic chlorine and more generally halogens do like to interact with Hc atoms which are hydrophobic and poorly polar (Jelsch *et al.*, 2015 ▸).

The ether O⋯Hc interaction, which is a weak hydrogen bond, is however more favorable than the Cl⋯Hc contacts. The average *E* values of the different interactions can be seen in Table 4 ▸. The O⋯Hc and Cl⋯Hc contacts are the only interactions which are enriched on average, suggesting that they are driving forces in the crystal packing formation while the hydrophobic C⋯Hc contacts with 〈*E*〉 = 1.00 (4) also have an important contribution. The competition between halogen bonding and hydrogen bonding has been investigated by Aakeröy *et al.* (2007 ▸).

The O⋯O contacts are the most avoided 〈*E*_OO_〉 = 0.11 (6) in this family of compounds, while Cl⋯O and Cl⋯Cl interactions are slightly less disfavored. The O⋯Cl halogen bonding occurs much more in molecules poor in hydrogen, in the case of chloro-ethers, as can be seen in Fig. S9, presumably because then O and Cl atoms are less likely to be all involved in hydrogen bonds. The enrichment of O⋯O and Cl⋯Cl contacts is shown in Fig. S10. Cl⋯Cl interactions are in chloro-ethers, on average, impoverished [〈*E*〉 = 0.55 (8)], but some compounds show incidentally high *E*(Cl,Cl) values reaching 2.57. The nature of C—Cl⋯Cl—C contacts has been investigated *via* charge density tools; in some geometric configurations, the interaction is attractive based on the polar flattening of the Cl atom electron density (Hathwar & Row, 2010 ▸).

As observed generally in oxygenated/halogenated hydrocarbon compounds, the Hc⋯Hc interaction is slightly disfavored (Jelsch *et al.*, 2014 ▸, 2015 ▸).

There are a few outliers to the global trends in Fig. 15 ▸. Compound TITQAO (C_17_H_10_Cl_10_O; Mackenzie *et al.*, 1996 ▸), which has ten Cl and only one O atom, has a peculiar chemical composition and forms no O⋯Hc interaction in the crystal packing. Instead it displays a Cl⋯O contact.

Short interactions between an oxygen and a chlorine atom are described in the literature as ‘halogen bonding’ (Metrangolo & Resnati, 2001 ▸; Politzer *et al.*, 2007 ▸) with the C—Cl⋯O angle larger than 140° (ideally close to 180°) and the Cl⋯O distance smaller than 3.3 Å, the sum of van der Waals radii (Wilcken *et al.*, 2013 ▸). The Cl⋯O contact in TITQAO crystal structure is a weak halogen bond with a short Cl⋯O distance of 3.129 Å, but C—Cl⋯O angle at 135.6°. FUDBAI compound (C_14_H_10_Cl_4_O; Etzkorn *et al.*, 2009 ▸, Fig. S11) with a low *E*(O,Hc) = 0.75 value is substituted with four chlorine and one oxygen atom; the latter atom forms an O⋯Cl halogen bond with *E*(O,Cl) = 1.34 and an O⋯H—C hydrogen bond. The C—Cl⋯O angle is 158.2° and the Cl⋯O distance is 3.041 Å.

Interestingly, in each of the two Cl⋯O contacts mentioned, the C—Cl bonds are oriented towards an electron lone pair of the O atom, then forming a favorable electrostatic interaction.

### 3.9. Chloro-ketones   

**Figure 16:**
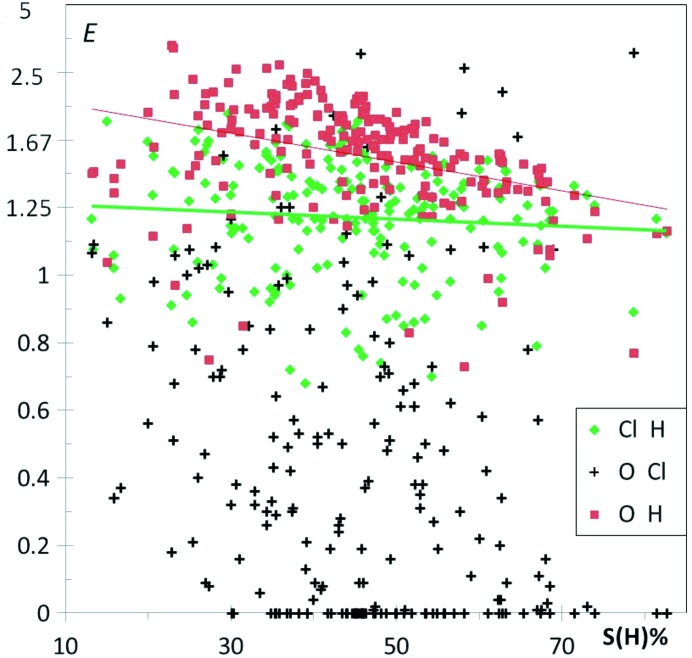
Enrichment of some contacts in hydrocarbons substituted with chlorine and ketone groups.

The behavior of hydrocarbons substituted with chlorine and with ketone chemical functions was explored from a series of 203 molecules retrieved from the CSD. The contact propensities as a function of H content on the molecular surface are displayed in Fig. 16 ▸. In this series, O⋯O contacts, as in most oxygenated hydrocarbons, are avoided with 〈*E*_OO_〉 = 0.06 (1). The contacts which are enriched are the O⋯Hc and Cl⋯Hc interactions with average *E* enrichment ratios of 1.55 (1) and 1.19 (1), respectively. These results, in concordance with the study on contact propensities in halogenated hydrocarbons (Jelsch *et al.*, 2015 ▸), confirm that oxygen is a stronger hydrogen-bond acceptor than organic chlorine.

The trends observed in Fig. 16 ▸ are similar to those found in ether-chlorinated compounds, but the O⋯H contacts are more favored in the ketone-chlorinated family. This behavior is in agreement with the fact that ketones are more electronegative and stronger hydrogen bond acceptors than ether O atoms, as already observed in Figs. 2 ▸ and 3 ▸.

While O⋯O contacts are avoided due to electrostatic repulsion (Fig. S12), the Cl⋯Cl contacts show a wide range of enrichment values between 0. and 2.6; chlorine is less electronegative and is considered as relatively hydrophobic. Halogen⋯halogen interactions have been described to show some directional preferences (Desiraju & Parthasarathy, 1989 ▸; Awwadi *et al.*, 2006 ▸). The presence of a positive electrostatic end cap on halogen atoms (except for fluorine) and of an equatorial torus shape electronic accumulation renders, in favorable orientations, the Cl⋯Cl interaction attractive from an electrostatic point of view.

**Figure 17:**
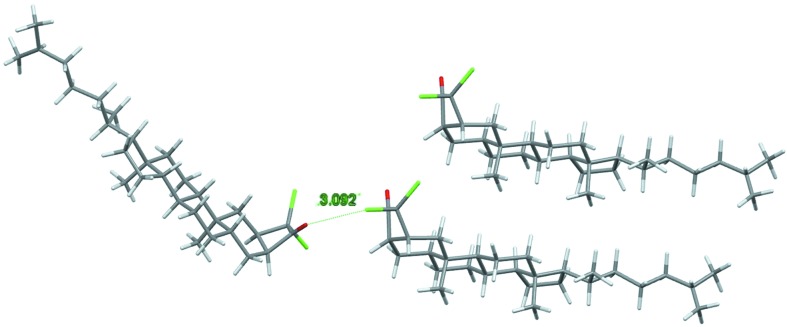
Molecule refcode COXBST is an example of an outlier among chloro-ketone hydrocarbons with a high hydrogen content and an elevated *E*(O,Cl) = 2.91 value. The crystal packing displays impoverished Cl⋯H (*E* = 0.89) and O⋯H (*E* = 0.77) hydrogen bonding while H⋯H (*E* = 1.04) contacts constitute a large part of the interactions.

Cl⋯O=c contacts are generally disfavored as 〈*E*(O,Cl)〉 = 0.44 (3), presumably due to electrostatic repulsion between two globally electronegative atoms. The Cl atom shows, however, an electropositive region along the C—Cl bond which allows the formation of favorable Cl⋯O interactions called ‘halogen bonding’ (Ding *et al.*, 2012 ▸; Sirimulla *et al.*, 2013 ▸). A minority of compounds have indeed high Cl⋯O enrichments ratios and in several molecules rich in hydrogen the Cl⋯O contacts are favored over the H⋯O weak hydrogen bonds. For instance, the chloro-ketone compound VIKLIK (C_29_H_26_Cl_2_O; Titouani *et al.*, 1991 ▸) displays such Cl⋯O halogen bonding and its *E*(Cl,O) value is 2.6. In addition to the Cl⋯O interaction, the unique O atom also forms two weak C—H⋯O hydrogen bonds (Fig. S13). Unlike chloro-ethers, some chloro-ketones with high hydrogen content also display enhanced Cl⋯O contacts (Figs. 16 ▸ and Fig. S9). This is, for instance, the case for the molecule with refcode COXBST (C_29_H_46_Cl_2_O; Nassimbeni *et al.*, 1977 ▸), which shows a close Cl⋯O contact and limited Cl⋯H—C and O⋯H—C contacts (Fig. 17 ▸). The Cl⋯O distances in VIKLIK and COXBST compounds are shorter than the sum of van der Waals radii, and the C—Cl⋯O angles are respectively 155.7 and 141.90°, so the latter halogen bond is therefore weak. The crystal packing of this compound may be governed by the hydrophobic forces involving the large aliphatic part of the molecule, as hydrogen constitutes 79% of the surface content and H⋯H interactions are enriched as they make up to 64% of the contact surface, with *E*_HH_ = 1.04.

### 3.10. Chloro-alcohol molecules   

**Figure 18:**
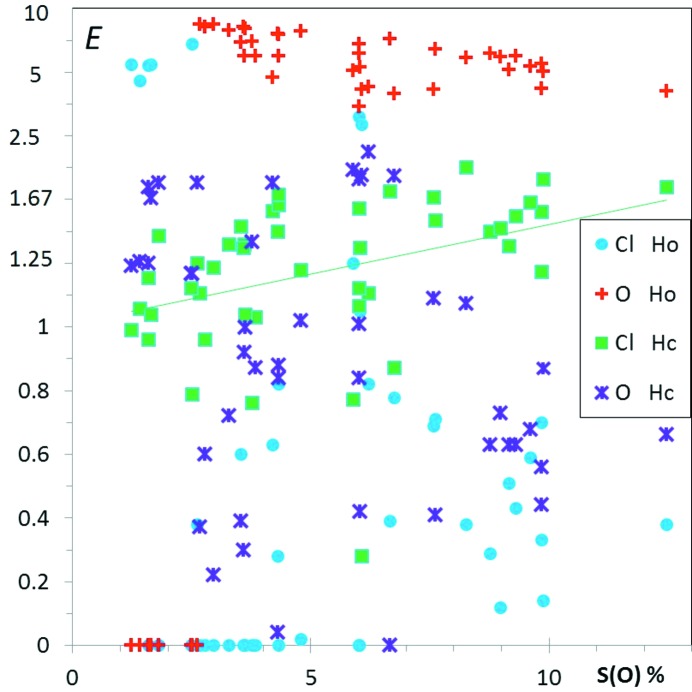
Enrichment of hydrogen contacts with oxygen and chlorine in chlorinated alcohols.

The study of chlorinated alcohols enables to compare the interaction behavior of oxygen and chlorine atoms with two types of H atoms, the hydrophobic Hc and the more electropositive Ho hydroxyl H atoms. In a previous study (Jelsch *et al.*, 2015 ▸), it was found that halogen atoms prefer to interact with Hc atoms while, on the other hand, O atoms have Ho atoms as a favorite partner to form strong hydrogen bonds. Both types of contacts can be considered as attractive electrostatic interactions between atoms of opposite charge, the former being however of more hydrophobic nature. The scatterplot of *E* values in Fig. 18 ▸ confirms globally these tendencies. For all compounds with at least 3% of oxygen on the surface, the O⋯Ho hydrogen bonds are strongly enriched with *E*(O,Ho) > 3. The more hydrophobic interactions Cl⋯Hc are generally moderately enriched with most *E* values larger than unity but below a 2.0 threshold. The interactions between pairs of weakly and strongly charged atoms such as Cl⋯Ho and Hc⋯O are, on the other hand, in general less favored than the strong hydrogen bonds O⋯Ho, but the two corresponding *E* ratios take a large range of values. The O⋯Hc contacts with 〈*E*〉 = 0.90 (6) can be impoverished or moderately enriched up to *E* = 2 values. The O—Ho⋯Cl interactions are very contrasted with enrichment ratios varying from 0 to 9.

**Figure 19:**
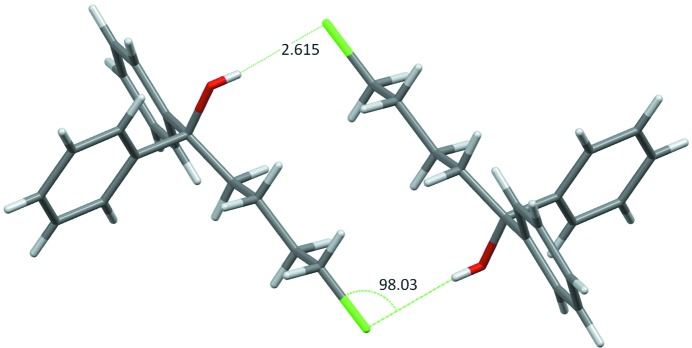
Example of an outlier among chloro-alcohol hydrocarbons with *E*(O,Ho) = 0. The crystal packing is devoid of strong O—H⋯O hydrogen bonds, but instead another less favorable electrostatic interaction takes place: O—H⋯Cl.

The scatterplot in Fig. 18 ▸ shows, however, a cluster of outliers with *E*(O,Ho) = 0 occurring for molecules with poor oxygen content. For some of these compounds, the absence of O—Ho⋯O hydrogen bonds is compensated by an O—Ho⋯Cl interaction which is a weaker hydrogen bond. The existence of intramolecular O—H⋯Cl—C interactions in several gem-alkynols has been reported by Banerjee *et al.* (2004 ▸). The C—Cl covalent bond and Cl⋯Ho contact directions are not far from being perpendicular in these structures. The molecule with refcode WECPIF (C_17_H_19_ClO; Ayala *et al.*, 2012 ▸) is exemplified in Fig. 19 ▸, which shows a dimer interacting through an O—H⋯Cl weak hydrogen bond, the C—Cl⋯Ho angle being 98.03°. The crystal structure of refcode WECPEB (C_15_H_15_C_l_O; Ayala *et al.*, 2012 ▸) is characterized by an absence of both O⋯Ho and Cl⋯Ho interactions, but an O—H⋯π weak hydrogen bond [*E*(C,Ho) = 2.56] is found instead (Fig. S14). This molecule with high content in C and H is presumably bulky with its two aromatic rings and cannot easily accommodate the formation of hydrogen bonds in the crystal. The enrichment of the other contact types for this family of molecules can be seen in Table 5 ▸ and Cl⋯Cl, Hc⋯Hc and O⋯Cl contacts are detailed in Fig. S15. Besides the O⋯Ho and Cl⋯Hc hydrogen bonds, the C⋯Hc hydrophobic contact is the only one to be enriched, on average. These three types of contacts presumably play a major role in the crystal packing formation of chlorinated alcohols.

## 4. Conclusion   

The interactions formed by several oxygenated chemical functions in crystal structures retrieved from the Cambridge Structural database have been compared. The Hirshfeld surface permits analyzing intermolecular interactions while maintaining a whole molecule approach. The contact enrichment methodology on families of compounds is a valuable tool to compare the likelihood of different contacts to occur. The analysis of contacts between chemical species which are subdivided in sub-groups yields indications on the relative strengths of the different interactions. The current study focuses on the interactions of several chemical types of O atoms and notably on their propensity to form weak and strong hydrogen bonds. For instance, in the ester hydrocarbon compounds, there are two types of O atoms. The enrichment of the weak C—H⋯O hydrogen bonds is, in general, higher for the O=c atoms than for the Occ oxygen atoms. This is in accordance with the fact that O=c oxygen atoms are stronger hydrogen acceptors than the Occ oxygen atoms.

The general principle ‘the best hydrogen-bond donor preferentially interacts with the best hydrogen-bond acceptor’ was explored by Aakeröy *et al.* (2003 ▸) and found to apply in the crystal structures of 12 salts based on asymmetric 2-aminopyrimidinium cations. From this principle, it can expected that C—H⋯O hydrogen bonds should occur more frequently when there is an absence or a deficit of strong hydrogen-bond donors, as they are not in competition with strong hydrogen bonds. The *E*(Hc,O) are indeed generally enriched in the case of ketones, ethers, nitro-ethers and esters (Figs. 2 ▸, 3 ▸, 4 ▸ and 5 ▸). On the contrary, for alcohols (Fig. 6 ▸) which have strong hydrogen-bond donors, the O⋯Hc interactions are generally under-represented. The case of phenols is more mitigated as strong hydrogen bonds sometimes may form only partly or not at all.

Phenols and alcohols are two hydroxyl groups which differ by the lower p*K_a_* of phenols which lose their proton in basic conditions (p*K_a_* ≃ 10). The behavior of the crystal interactions of these two kinds of hydroxyl groups have been analyzed accordingly. Since a significant number of carbohydrates substituted with both alcohol and phenol group were found in the CSD, the mutual interactions between the two chemical functions could also be observed together. Globally, for the class of combined alcohol/phenol molecules, the crystal packing is governed by strong hydrogen bonds O—H⋯O along with C⋯Hc hydrophobic interactions. However, the current study also highlights some hierarchy between the contacts with more fine differences between alcohols and phenols which prefer to form mutual cross hydrogen-bonding, rather than to interact with themselves. The most favored hydrogen bonds are Hp⋯Oa cross contacts between the hydroxyl hydrogen of phenols and oxygen acceptors of alcohols. From a chemical point of view, the phenol Hp atoms are the most polar among the alcohol and phenol hydroxyl atoms, therefore finding oxygen acceptor partners to Hp atoms is a priority in the crystal assembly process of these compounds. The study also highlights that when no oxygen acceptor is available to form a hydrogen bond with a hydroxyl hydrogen-bond donor, sometimes a π aromatic system plays the role of acceptor.

Analysis of contacts in crystals of oxygenated hydrocarbon compounds has permitted to highlight the changes brought by inclusion of water in crystals on the interaction networks of host molecules. Indeed, strong interactions such as hydrogen bonds between host molecules, although existing, are superseded by cross contacts between water and functional groups of host molecules which are the most enriched. Consequently, in monohydrate crystals the interactions of water ensure cohesion and stability of crystal packing by maximizing the number of hydrogen-bond donors and acceptors which have a partner.

The study also highlights the deposition in the CSD of a non-negligible part of structures with some obvious errors which can be detected by the analysis of the contacts through the Hirshfeld surface methodology. This is particularly the case for C—O—H hydroxyl groups which have a rotational degree of freedom. The wrong positioning of hydroxyl O atoms can result in O⋯O and O—H⋯H—O contacts which are unfavorable from an electrostatic perspective and which present some atom bumping problems, in addition. The more systematic application of crystal structure validation software such as *checkCIF* (Spek, 2003 ▸) would be of great benefit to identify such errors, notably for crystal structures published in chemistry journals.

The article gives a few examples of multiple linear regressions which are directions to further explore in future investigations of crystal packing contacts in conjunction with Hirshfeld surface tools. Many descriptors such as the size/shape of the molecule, electrostatic moments, molecular symmetry, geometric distribution of chemical groups *etc.* could be incorporated into the modeling, in addition, to the chemical proportions of the molecule.

The *E* value cannot always be considered as a statistic as it results sometimes from a single observation, in the case of molecules where there is only one copy of a chemical type. The *E* values can tend to take either zero or high *E* values when the molecule contains only one substituent. This is the case for moderately enriched or disfavored contacts, such as O⋯Cl contacts in chloro-ketones and chloro-alcohols (Figs. 16 ▸ and 18 ▸). This also occurs in cases when there are many chemical sub-types such as in Fig. 9 ▸(*a*), where six different types of O⋯H contacts are analyzed in alcohol–phenol hydrocarbons.

Then it is more pertinent to look at trends on a series of molecules. However, contacts which are strongly avoided (such as O⋯O) or favored (such as O⋯Ho) will generally still follow a general trend even if only one copy of the chemical types is present on the molecule. This applies for instance in molecules poor in oxygen for Hc⋯O contacts in the case of ketones (Fig. 2 ▸), ethers (Fig. 3 ▸), esters (Fig. 5 ▸) and Ho⋯O contacts in the case of alcohols (Fig. 6 ▸) or even O(water)⋯H(alcohol) in alcohol monohydrates (Fig. 13 ▸).

In addition, single observations are still worth looking at as a peculiar high or zero *E* value for a given contact can often find an explanation in the crystal packing itself or from the molecular geometry/shape. There are several cases of phenols with low oxygen content and zero *E*_HoO_ values; the absence of intermolecular strong hydrogen bonds is then due to steric hindrance or to the occurrence of intramolecular hydrogen bonds or O—H⋯π interactions instead.

The statistical analysis of crystal contacts could also take into account the influence of space group and molecular chirality. For instance, symmetry operations in the crystal such as mirrors and, to a lesser extent, rotation axes, should yield geometrically larger occurrences of self-contacts, which are generally not favorable from an electrostatic point of view, except for hydrophobic interactions. Other symmetry operators such as helical axes and glide planes are generally preferred as they make different parts of the molecule interact with each other, as confirmed by statistics on space-group frequencies in organic crystals (Mighell *et al.*, 1983 ▸).

While a significant share of contacts in organic crystal structures can be classified in structural motifs such as hydrogen bonds or aromatic stacking, a part of the cohesive potential energy in organic crystals is stored in structurally non-specific molecular contacts (Dunitz & Gavezzotti, 2005 ▸). While the strong and weak hydrogen bonds appear favored in crystal structures according to their *E*_HO_ > 1 enrichment values, the latter contacts, which may include hydrophobic interactions, should yield enrichment ratios which are moderately enriched or impoverished, depending on the crystal structure context.

The study of interaction propensities of chemical functions in crystal packing constitutes an effective tool in identifying the presence or absence of significant structure-directing factors. Furthermore, the strategies that underlie crystal-structure prediction (CSP) methodologies (Price, 2014 ▸) are based partly on already known structural features. Therefore, knowledge of interaction propensities of chemical functions in crystal packing constitutes an effective precursor filter to the computationally intensive polymorph-prediction methods. Interaction propensities between chemical groups and hierarchies can also be applied in the design of co-crystallization experiments to obtain pharmaceutical co-crystals of drug substances (Duggirala *et al.*, 2016 ▸) with improved physicochemical and clinical properties.

## Supplementary Material

Additional enrichment ratio figures, list of molecules kept and deleted, examples of peculiar crystal packing. DOI: 10.1107/S2052252516020200/zx5008sup1.pdf

## Figures and Tables

**Table 1:** Linear regressions of the 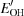 values in ketones as a function of chemical content and molecular weight *W* *E*° is the ordinate value at the origin. For instance, the first row equation is *E*_lr_ = 1.06 × *S*(O) + 1.01.

*W*	*S*(C)	*S*(H)	*S*(O)	*E*°	Correlation
			1.06	1.01	0.451
	1.05			1.01	0.451
		−0.74		1.72	0.523
		−0.65	0.17	1.62	0.526
9.6E-05		−0.64	0.16	1.63	0.526

**Table 2:** Average and sample standard deviation (s.s.d.) enrichment ratios in the crystals with *Z*′ = 2 of alcohol–ketone molecules The *c* correlation coefficients were computed between the two sets of values (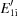, 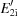) and (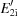, 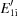), *i* = 1⋯17; this way *c* is independent of the attributions of dimers #1 and #2.

Contact	O⋯O	Ho⋯Ho	Hc⋯Hc	C⋯C	O⋯Ho	O⋯Hc	Hc⋯Ho	C⋯Ho	C⋯Hc	C⋯O
〈*E*〉	0.12	0.69	1.02	0.99	3.41	0.99	0.65	0.36	1.06	0.47
s.s.d.(*E*′)	0.19	0.62	0.08	0.41	0.14	0.18	0.22	0.31	0.17	0.33
Correlation	0.620	0.800	0.615	0.534	0.770	0.376	0.472	0.770	0.825	0.879

**Table 3:** Average *E* and s.s.d. of *E*′ values in alcohol–ketone monohydrates

Contact	Ha⋯Oa	Hw⋯Oa	Ha⋯Ow	Hw⋯Ow	Ha⋯O=c	Hw⋯O=c	Hc⋯O=c	Hc⋯Oa	Hc⋯Ow
〈*E*〉	0.62	1.87	7.11	0.13	0.47	2.12	0.95	0.83	0.83
s.s.d.(*E*′)	0.82	0.63	0.06	0.40	0.77	0.52	0.24	0.36	0.27

**Table 4:** Average *E* and standard deviation of *E*′ values for the different interactions in chlorine/ether compounds

Contact	Cl⋯Cl	Cl⋯Hc	Hc⋯Hc	C⋯Cl	C⋯Hc	C⋯C	O⋯Cl	O⋯Hc	O⋯C	O⋯O
〈*E*〉	0.55	1.20	0.84	0.95	1.00	0.77	0.40	1.32	0.35	0.11
s.s.d.(*E*′)	0.44	0.10	0.10	0.35	0.25	0.44	0.46	0.28	0.43	0.35

**Table:** 

Contact	Cl⋯Cl	C⋯C	O⋯O	Hc⋯Hc	Ho⋯o	Hc⋯Ho
〈*E*〉	0.56	0.82	0.06	0.75	0.49	0.60
s.s.d.(*E*′)	0.53	0.49	0.09	0.29	0.60	0.28

**Table:** 

Contact	Cl⋯Ho	Cl⋯Hc	Cl⋯C	Cl⋯O	C⋯O	C⋯Ho	C⋯Hc	O⋯Ho	O⋯Hc
〈*E*〉	0.55	1.21	0.85	0.17	0.63	0.64	1.08	1.91	0.90
s.s.d.(*E*′)	0.62	0.23	0.36	0.26	2.67	0.54	0.25	0.75	0.43
