# Supplementary material for: Atom interaction propensities of oxygenated chemical functions in crystal packings
Source: IUCrJ. 2017 Jan 21;4(Pt 2):158–74. doi: 10.1107/S2052252516020200 (PMC5330527; doi:10.1107/S2052252516020200)
Supplement: Supplementary file 1 [file m-04-00158-sup1.pdf]

IUCrJ

Volume 4 (2017)

Supporting information for article:

Atom interaction propensities of oxygenated chemical functions in crystal packings

Christian Jelsch and Yvon Bibila Mayaya Bisseyou

Supplementary Materials.

Atom interaction propensities of oxygenated chemical functions in crystal packings.

IUCr J.

Christian Jelsch[a] & Yvon Bibila Mayaya Bisseyou[b].

[a]CRM2, CNRS, Institut Jean Barriol, Université de Lorraine, Vandoeuvre les Nancy cedex, France,

[b] Laboratoire de Cristallographie et Physique Moléculaire, UFR SSMT, Université Félix Houphouët-Boigny, 22 BP 582 Abidjan 22, Côte d'Ivoire.

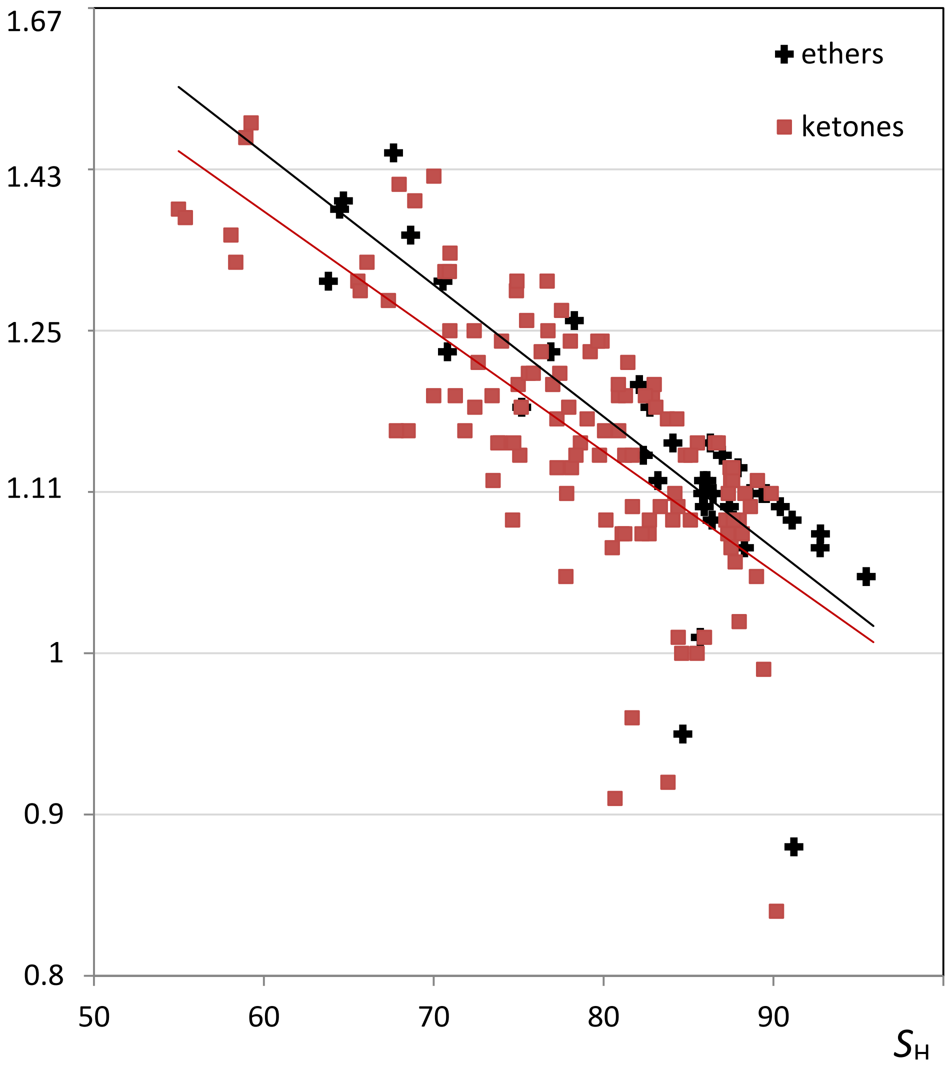

Figure SUP1. Comparison of enrichment ratios E_{OH} for ketones and ethers as a function of hydrogen content on the surface.

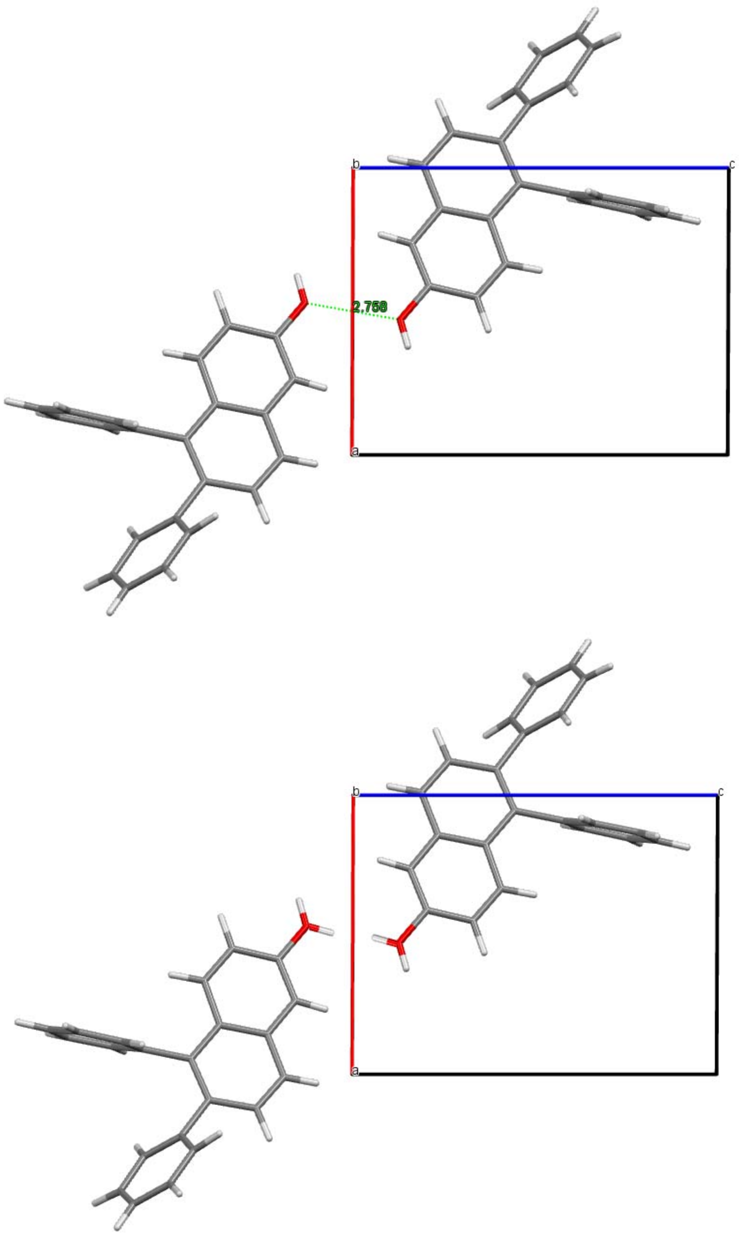

Figure Sup2

View of a dimer of KAZHAU molecule, which is an outlier among phenols with a large *E*(O,O) =11.8 enrichment ratio. The middle of the O…O short interaction is an inversion center. Top view: the published structure. Bottom view: proposed correct structure with a disordered hydroxyl hydrogen atom forming a O-Ho…O hydrogen bond (occupancies ½). Reference: Stanciu, C., Olmstead, M. M., Phillips, A.D., Stender, M. & Power, P. P., (2003), *Eur. J. Inorg. Chem.,* 3495-3500.

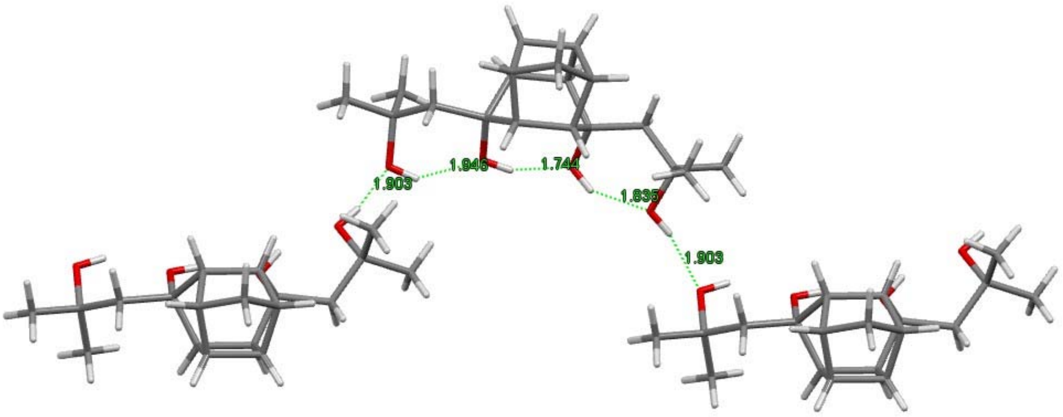

Figure SUP3

Example of an alcohol with an only moderately enriched O…Ho contacts which appears as an outlier in the [S_O,E(O,HO)] scatterplot at position S_O=7.6 and E(O,Ho)=2.25. The molecule is peculiar at it has three intramolecular H-bonds.

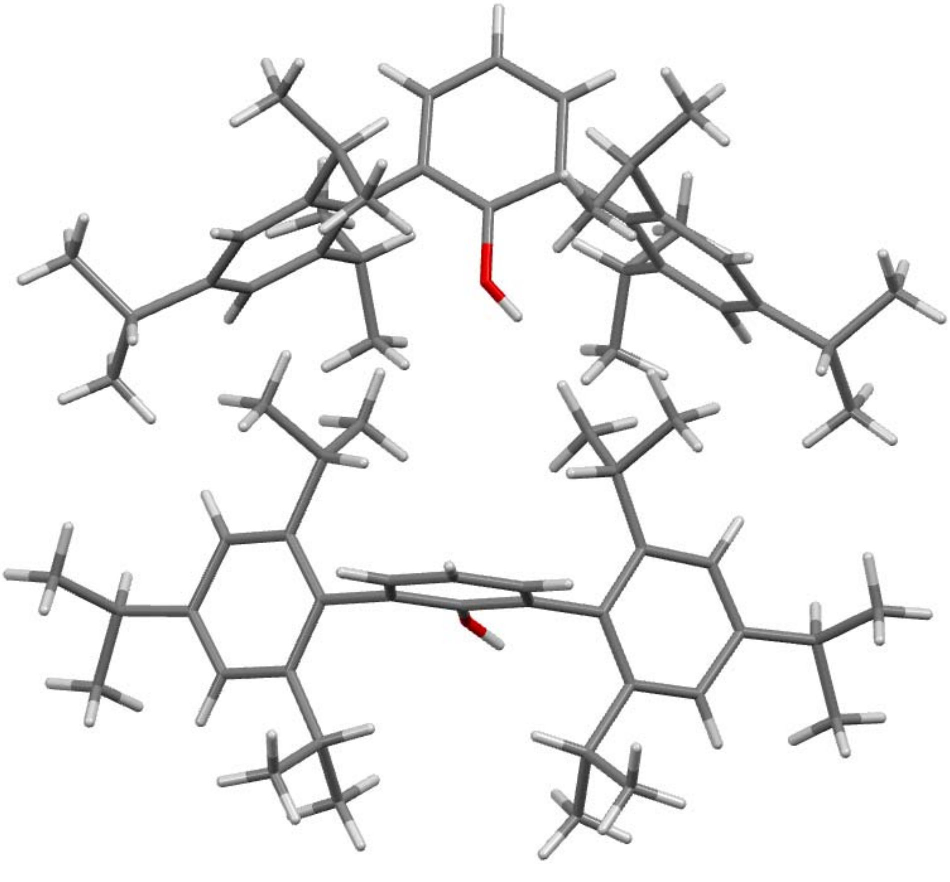

Figure Sup4.

Exemple of an outlier phenol molecule (refcode AKUSEE) without intermolecular O-H…O hydrogen bond due to the bulky environment around the hydroxyl group (view of a dimer).

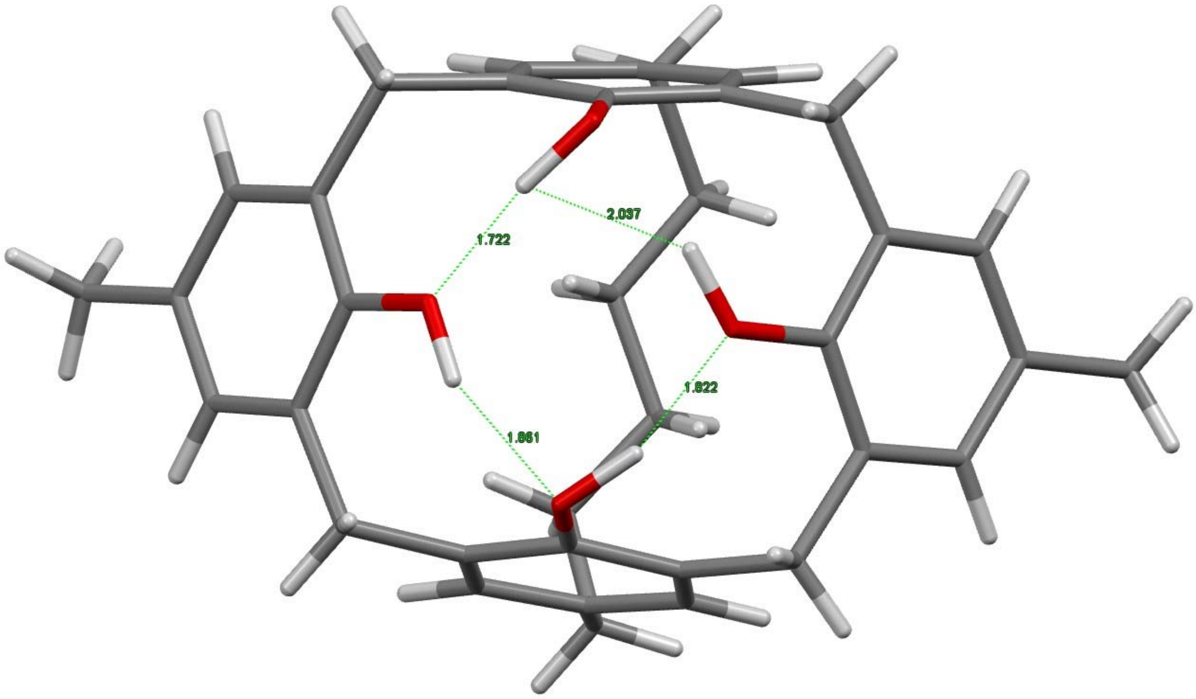

Figure Sup5.

Example of an outlier phenol molecule (refcode JABVUD) without intermolecular O-H…O hydrogen bond but with intramolecular H-bonds.

Figure Sup6.

Example of an outlier phenol molecule (refcode TUJYAZ) without intermolecular O-H…O hydrogen bond but with intramolecular C-H…π hydrogen bonds

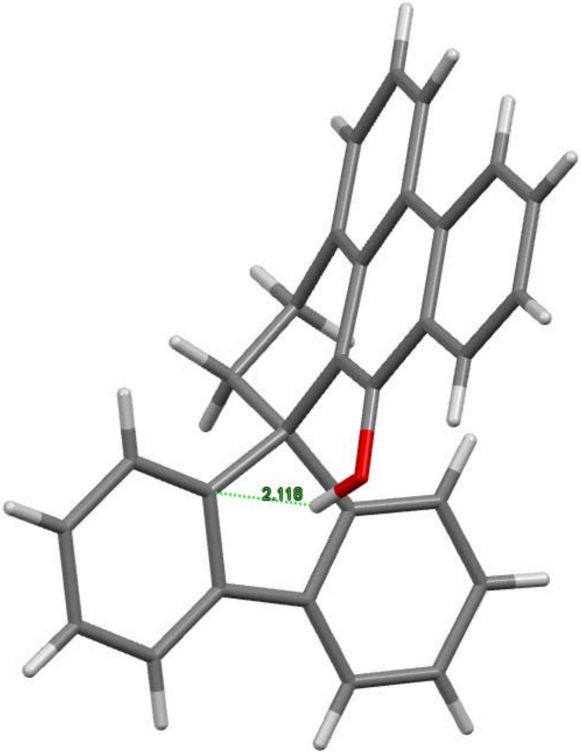

Figure Sup7.

Repartition of Hirshfeld surface content as function of S_O oxygen content in phenols.

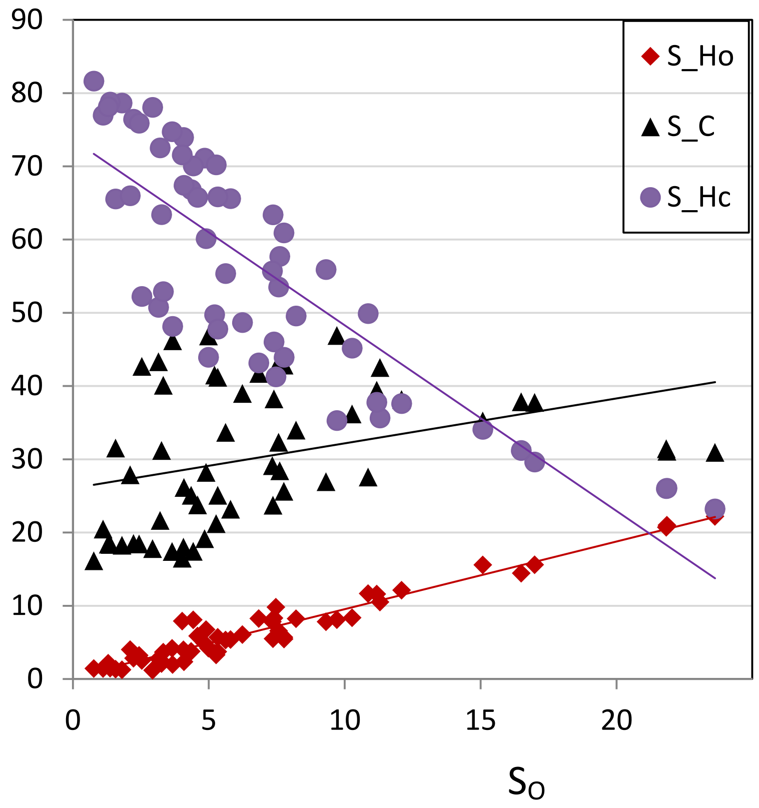

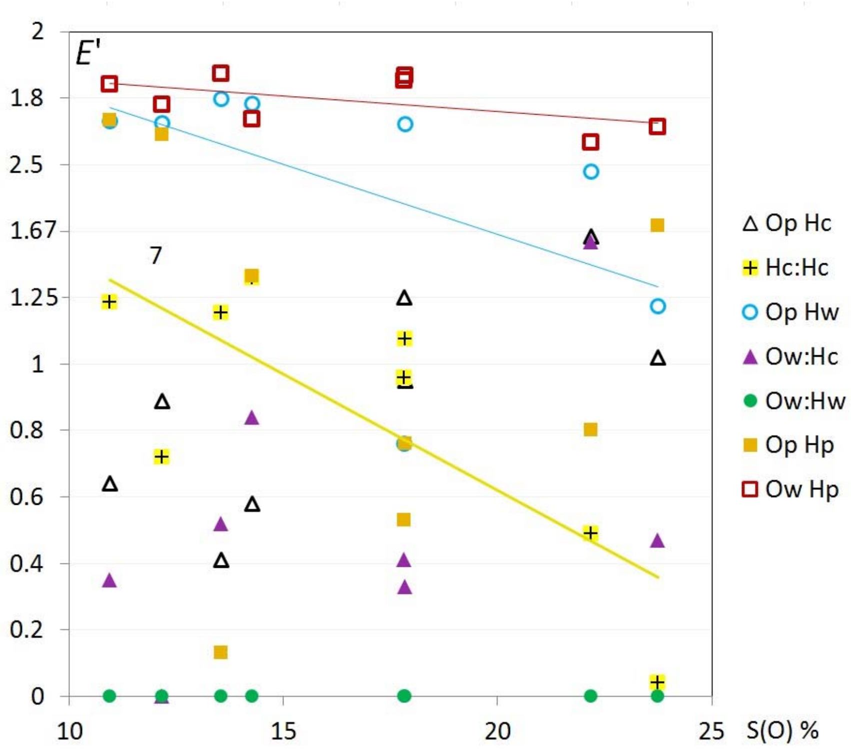

Figure Sup 8. Enrichment of the oxygen…hydrogen contacts in crystals of phenol-monohydrates as a function of total oxygen content on the molecular surface. "p" and "w" suffixes refer to phenol and water, respectively. The *E* ratios of the Hc…Hc hydrophobic interactions are also displayed.

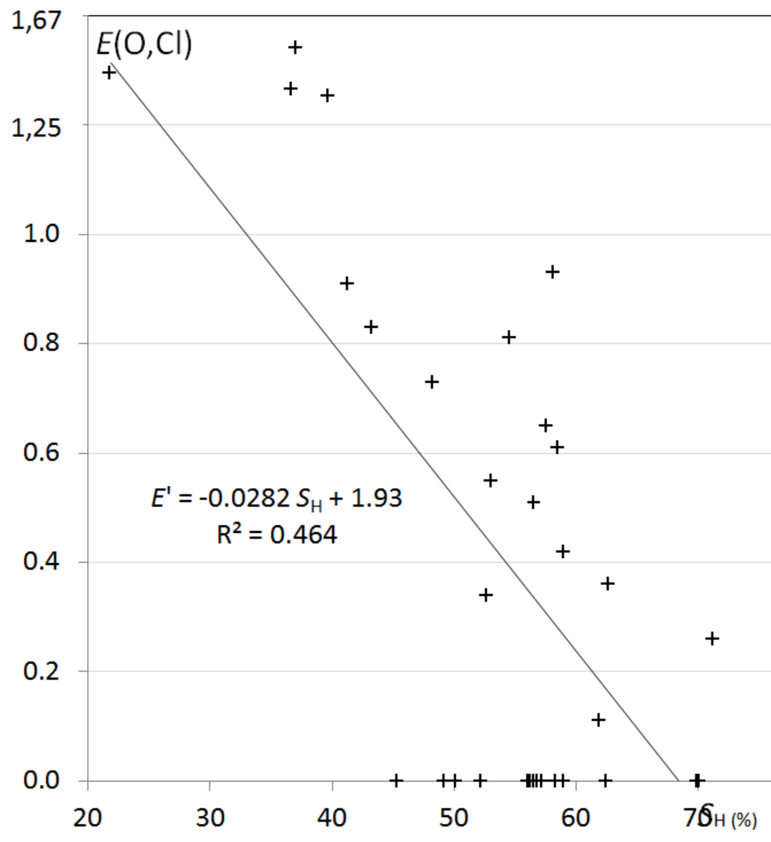

Fig. Sup9. Enrichment E(O,Cl) in chloro-ethers as a function of S_H.

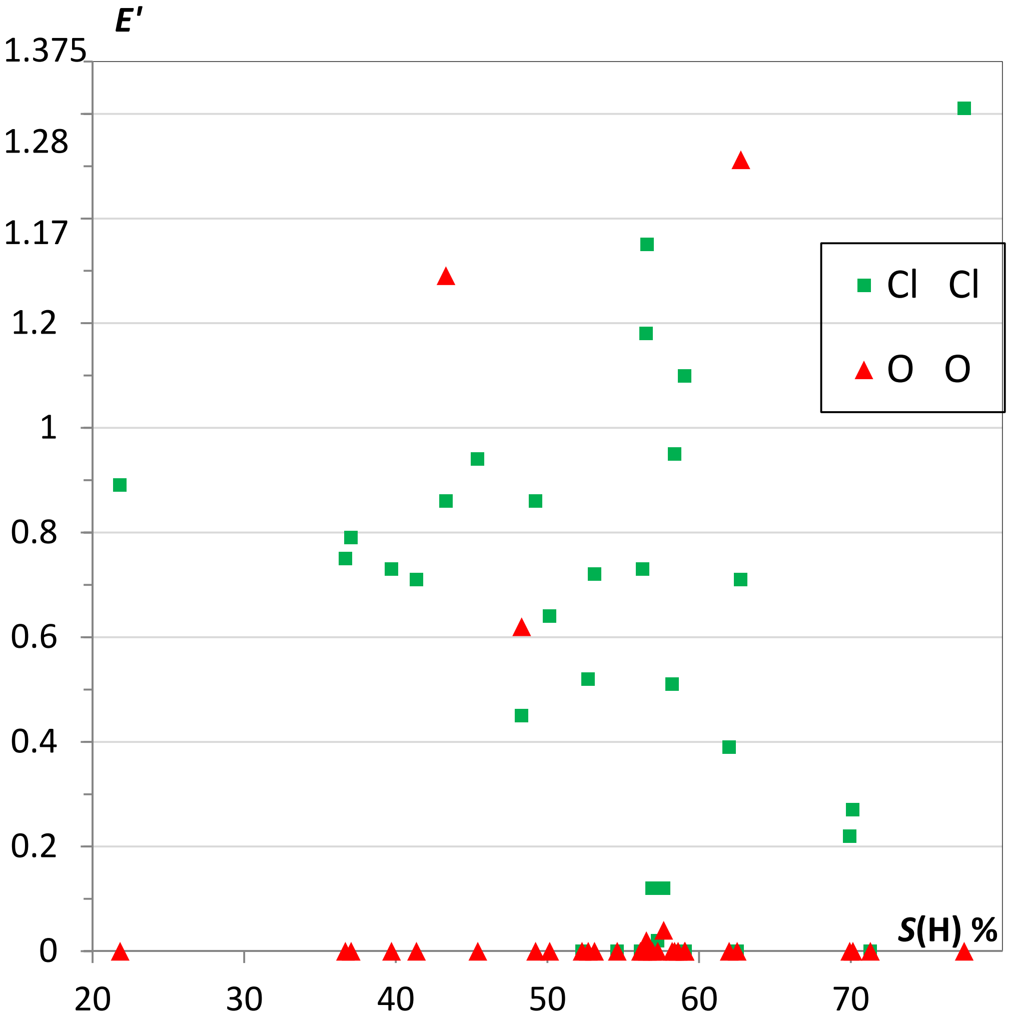

Figure Sup10. Enrichment ratios between chlorine and oxygen atoms in chloro hydrocarbons containing an ether group.

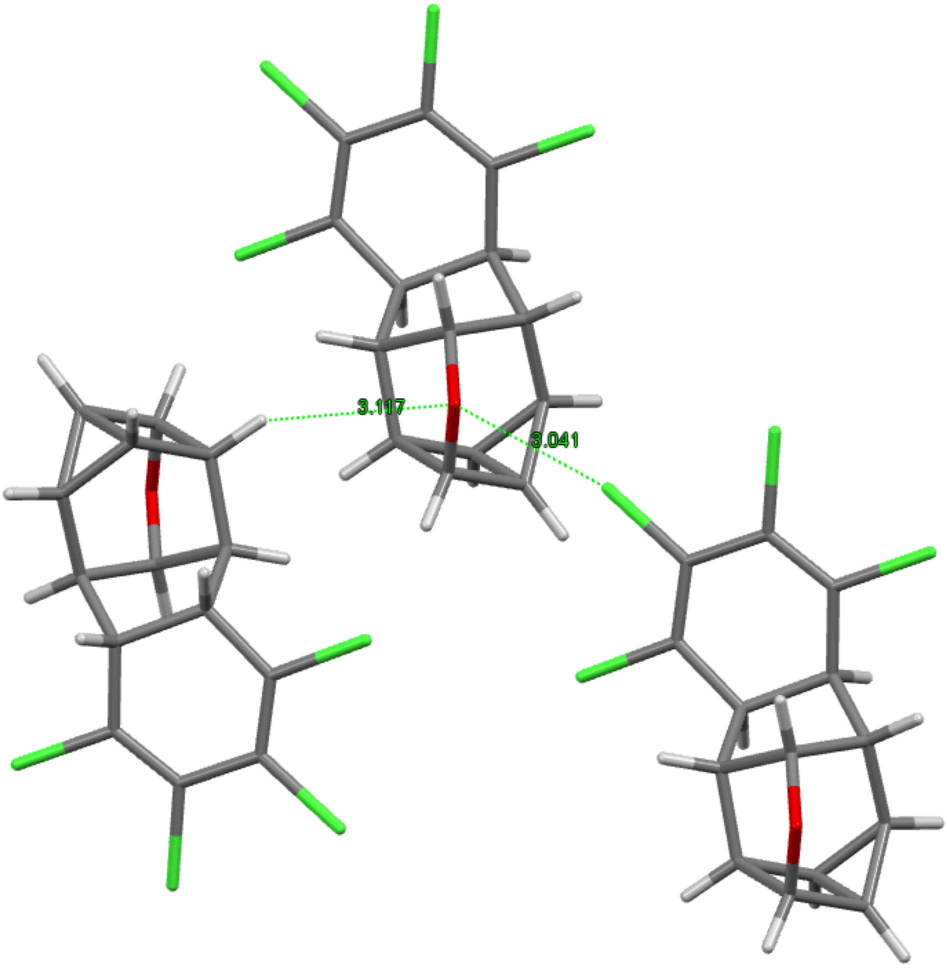

Figure Sup11 Example of a chloro-ether compound with high *E*(O,Cl)= 1.34 . The unique ether oxygen atoms forms a O…Cl halogen bond and a C-H…O weak hydrogen bond. The angle C-Cl…O is 158.2°. Compound refcode FUDBAI: Etzkorn, M., Amado-Sierra, M. del R. I., Smeltz, S. D., Gerken, M. (2009), *Tetrahedron Lett.* 50, 2991-2993.

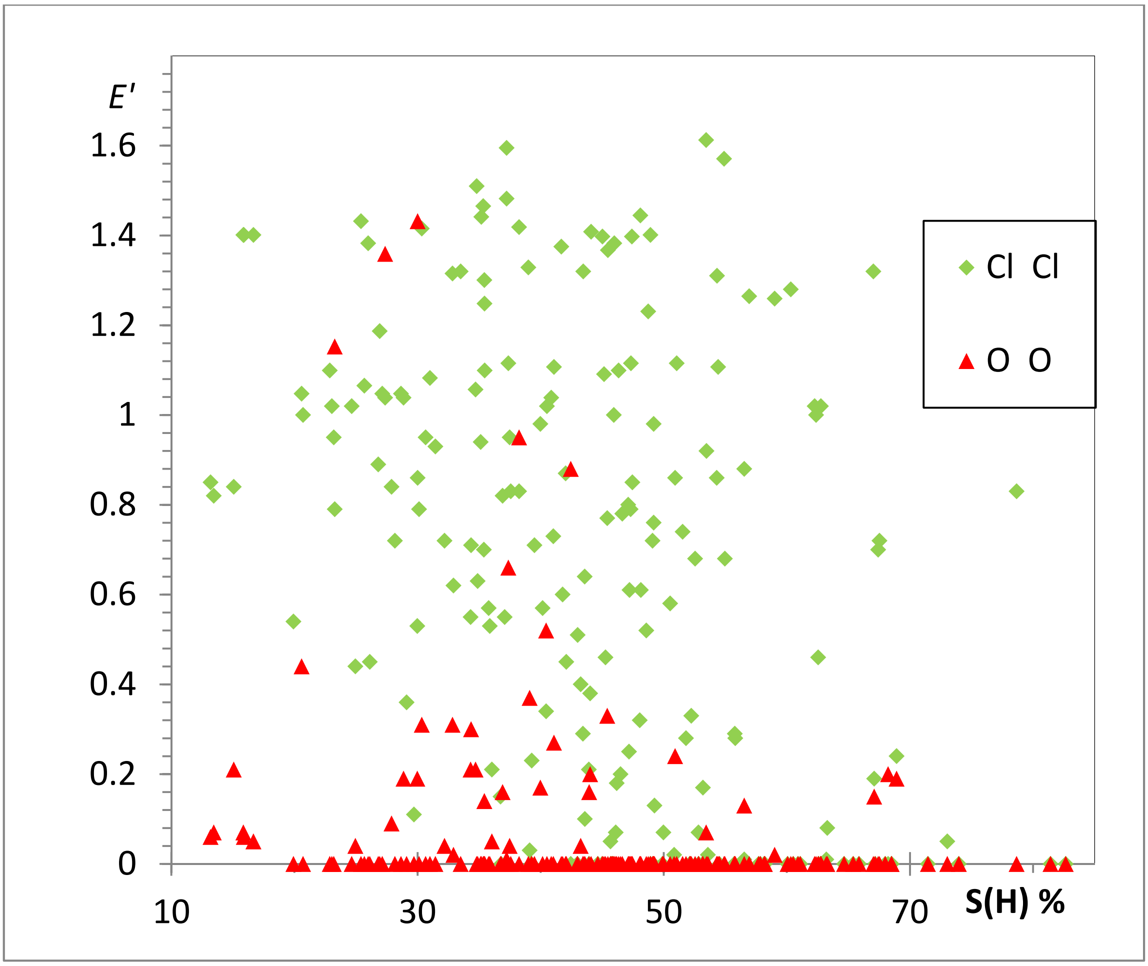

Figure Sup12.

Enrichment ratios between of chlorine…chlorine and of oxygen…oxygen contacts in chlorinated ketones. .

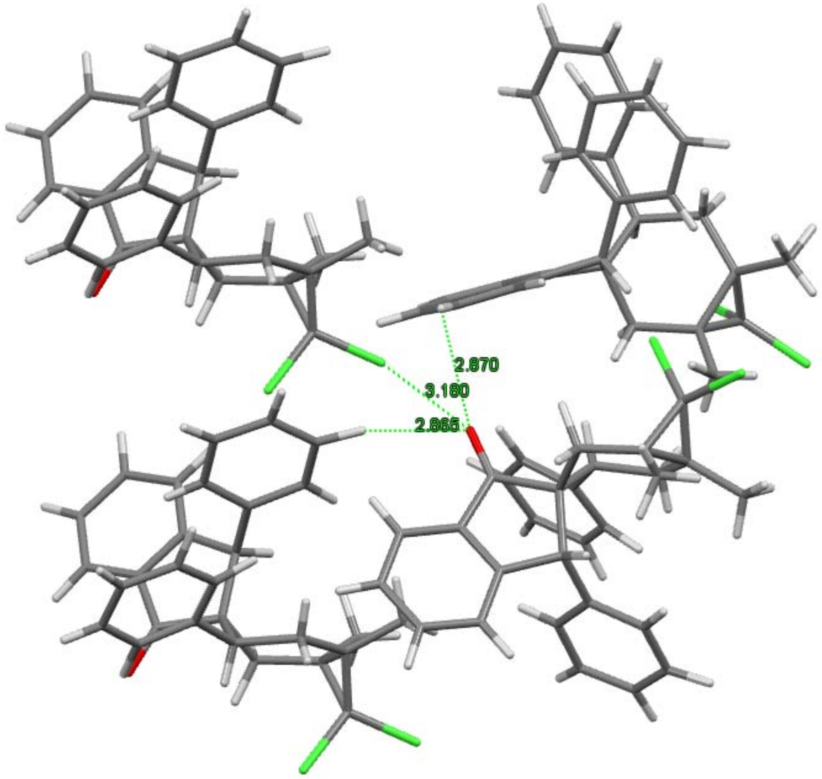

Figure Sup13

Example of a chloro-ketone compound VIKLIK with high *E*(O,Cl)=2.58 . The unique oxygen atom forms an O…Cl halogen bond and two C-H…O weak hydrogen bonds. The angle C-Cl...O is 155.7°.

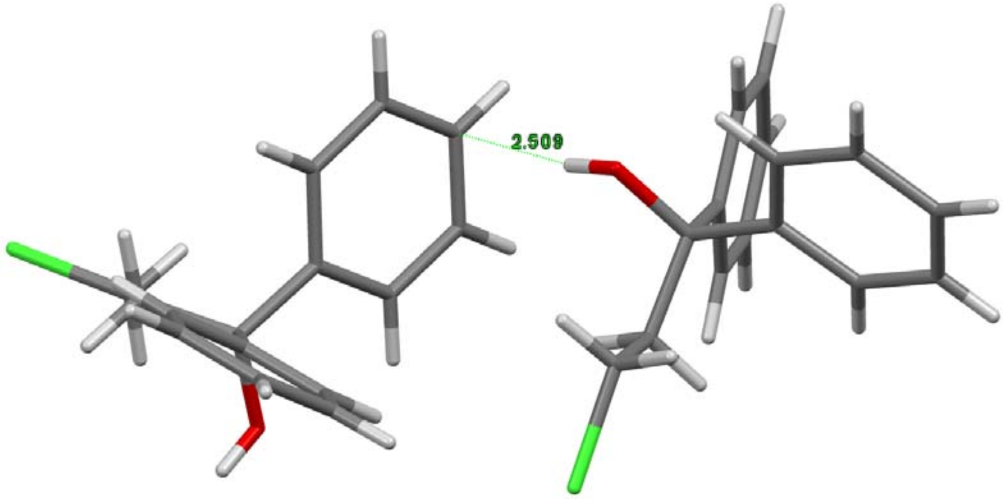

Figure Sup14. Example of an outlier among chloro-alcohol compounds with absence of strong hydrogen O-H…O and of O-H…Cl contact. The unique hydroxyl hydrogen atoms forms an O-H…π hydrogen bond with a phenyl ring.

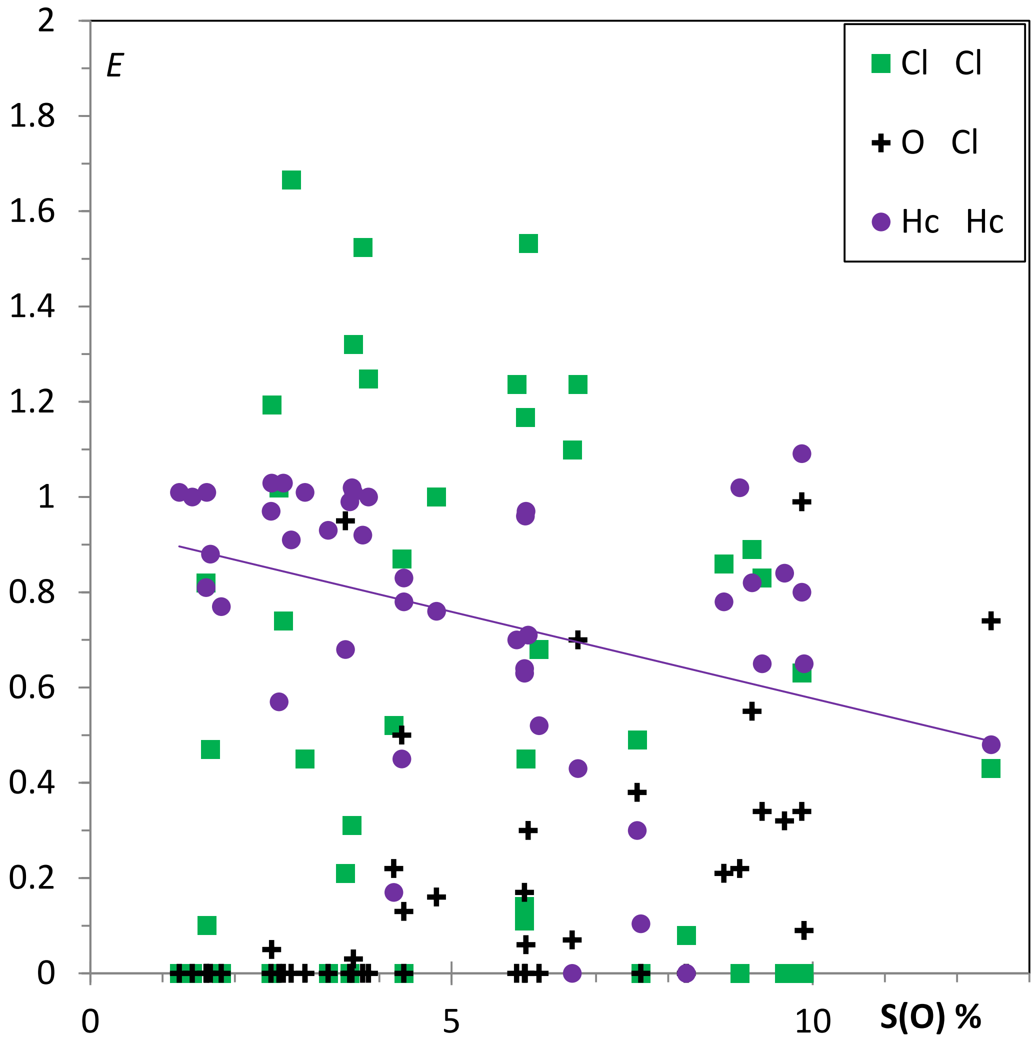

Figure Sup15

Enrichment ratios of some contact types in chloro-alcohols.

Table SUP1

List of oxygenated hydrocarbon compounds retrieved from the Cambridge Structural Database for each family of analyzed molecules.

LIST of ketones

ADETAE ANDION11 ATCDEO BEHZIX BEHZOD BNORSO10 BOJVUS BUCHAI CASJUB CEDDUK01 CEVVOO CEVXAC CIFSOB COQCUG CUFHAM CUJXOU CUJXUA CUJYAH CYDECO DANJEH DAZVEF DELZID DELZOJ DIWLOK DIWMEB DIWPII DIWPOO DUSDOL DUWBEC EBAVAE EJEFOO FONCOB FURGII10 FURGOO10 GEBRAG GETNUO GILZUW HABZAL HEBBEV HEJGUY HIXHIF05 HMTCDO INUHII JAPBIL JAPBOR JAPDAF JIBFOP JOPLOP JULJUV KEPBAJ KETTUY01 KETVAG10 KIKYOS KIMBOX KOQHON LAVDAN LAVHIZ LAVHOF LAVHUL LIHMIA LIHMOG LIXMOV LIZNOY LIZNUE LULHIJ MABEST MDHPNO MICRAT03 MICRAT04 MUSMUI NAFBOL NCUBEB10 NOYWAZ OFOQIL OMCHDO OXCHHX PADCAY PCDODO PROPTO PUKJUA PUSDEM QEYFAB QIFHESQOYTAZ QULNIV QULYOL RASWEN RIJGAS ROCMAY SAZVAQ SEJFOC SEJPOM SIHXUE SIHYAL SILMIJ TABNON TBUCHO TCDODO TCPDON TCTDTO20 TETMOU TETNIP TETNIP01 TMCYNO TOHZUL UNDECO UYUGAX VAMKEZ01 VARCUM VAXRES VOQRAU WIHJAZ XEQWOG XONXON YABCOT ZAKNUU ZZZMZM01 ZZZQAI02 ZZZWGK01

LIST of aliphatic ethers

CANNIO CEZSEF CMPANL01 CUKCIU01 DCHXCR01 DCHXCS01 DCHXCS02 DCHXCT DCHXCT01 FOBHIN GASLUI GEFJIK GEFJOQ HAZKAV HOXOCD02 LAKDEH MECZID MOFPAY NABREN NAKWUR OBIBOQ PUTHOB QEGSOL SEMKOK SIPSAL SISMUC SISNAJ TANMAT TEMCAP TIVHOW TIVJAK TIVJOY TIVJUE TOXCDP TOXCTD VEQKEH ZZZWGK03 YAGCEQ ZONRAV ZOSSEF

LIST of esters

ADUQIZ AGAJEX AGAJIB ALUVIM BEDNAZ BEFJUR BEKJIK BOBCIE BOBCIE10 BOJZUV BOPVIN BOSPUO CBTCME CINGAH CMEOXE01 CORNIH CORZAK DADQIK DEVBIP DODCHD DOJFOZ DOJFUF DOJGAM DUFMIA ECOTDA FAFDAS FAFDAS01 FAFDAT FATRUN FAWLEU FEJKOV FEMLEO FICYUM FITGEU FOLVUX FUMJON GEJLAK GEQQIC GOGXUV GOKMOK HEGSAN01 HEGSAN10 HEMKOZ HIFWOI HINHUJ HINJEV HOSNAE HOYTOF IWUFAH JAXMIF JEDZAT JEGYIE JIRXIS JORJEF JORJEF01 JORSIS KAMRAS KAXPOP KEJXII KEJXOO KEMFOY KUZXIN LEDNOX LEDPIT LITLIK MAJYAX MAMNAC MCMSCA10 MESQOR MTCDEC NACCID NETDOG NIFCOU NIFCUA NOSLOW NUQPIZ OMIDOD PAPVOR PATCUI PEHWAA PEKKEV PERCEN PESCUL PIJQOO PILLEB PIYYIG QETVUG QIKBER QOYKIY SEDLUI SEJWOT SIQQEP SUKXAX SUNHIS SURCEN SURCIR TELREH TIHMUS TIHNAZ TIVCOR TORMAP UCOJOM VANLEB VARPEJ VEBYEG01 VESCAY VESDAZ VEXRAR VODFOJ VOHNAH VUPVOR WECCAI WECCUC01 WELSUD WIRPAO XAGKEW XEGKID XODHAZ YARZUN03 YIXTUV ZAMVAK ZILQAM ZORKUM

LIST of nitro ether

ACENUS BAWJIT DADBAM DARGAF EFAXOY FALHUW FEFPEM FESPEY IPATOJ JUKSIR JULGAY MEMDIS MUBLUR NILNOL NNAPMP NUBZIU QEDYOP RUJBAA RUJSEV SILRUB SINJIJ VALTEJ YAWPAP ZIKYIB

LIST of alcohols

ACAVIJ AFILEI ASOCER ASOCIV ASOCOB ASOTOR BAJNAB BAVLOZ BCHMOL BESWON BEVBAF BILMIU BOGKIR BOMKUJ CAVDEI1 CEJXIY CERMOB CERMUH COYFEB CUVFII CYOCDL01 DABKOG ECEPEJ EHIXOI FECCOF01 FEPKUG FERTAY FEXKOI FIFRIV01 FUNCIA GIGYAW GOJKEV GOMQUV GOXMAH GUHBIU GURBUQ HANPAN HEBDIB HIYHAY HOCCAD HUKLIJ HXCHEX ICAHID IQAFAI JAJSOC JAXLUQ JEDBUQ JEQZUB JONLIH JONLON KAPNAQ KEKPEW KEPSON KEZTIU KEZTOA KOFFUH KOPKUV KUKYAR KUXFIT LOBQOK LOJXIS LONGAX LUDTUA MAMGEN MATLIC MEGJAJ MEGJEN MENVIL MINFEV MURABD MUTYOP NAYZOD NEPGCL01 NEZFON NIWNUC NULZEA NUMTUK PELBIR PERYTO04 PICYAB PILRAE PIMARB PIRBOJ PIRLOT PIWXIK PIYXOL PMGLYC POHYEQ POJGUQ POJYIX POMWUK PUFJIJ QATTEK QATTOU QATVEM QAZQAJ QIMCUK QURRUQ QUTFOA RAJDIP RIHMAX RIQMAG RIWTIB01 RIYZEF ROGNIL RUKNER RUSKEW RUVWAG SEVPAL SEVPEP SILGEZ SIQGUU SUPMIA SUPMOG TEZQIA THXCHO10 TIQHAC TIXXED UDEMEV VAHXAD VEKMED VIDXIR VIWHAL VIWTAX WEFWAH WEJMAA WERTUI WESTET WESTIX01 WOYKOK WUTMAA XEWKER XEYMIX XIDQUW XIJPEL YAJYIS YEJNIK YEYLUK ZEFPAD ZINWAU ZZZFFY01 ZZZGSC01 ZZZKPE01 ZZZPSA01

Alcohols molecules discarded, due to wrong hydroxyl orientation.

Detected by high *E*(O,O)

BCUNDL CUVFII EHIYEZ GUHBIU GEBSUB NEMXUY QIVYIF
RIQMAG SETJIK SOTBEI TEZQIA UDEMEV VIDXIR

Detected by low *E*(O,Ho)

EPFRED02 SETJIK EHIYEZ NEMXUY SOTBEI QIVYIF

LIST of phenols

ABUCOP AXEKOD BECNII BECPAC BEZRIJ BIRKOC01 CARJIQ CARKOX CATCOL12 CEYKUM CONNID CUFVII CUPDUM DENSOL01 DOGWOL DOGWUR DOHDPH02 EJEVOE ESTILO03 FEVCAL FIPYAG GALCAY HEGFAB HESKOF HESKOF01 HYQUIN08 ILABAZ JABWAK KAPVIH LERFET01 LESVEL MEBMIP NAPHOL01 NIYQUH NIYRAO NPHLDL01 NUTSUQ OTASUY PHGLOL01 PIDZIM PYRGAL02 PYRGAL03 RAGLEQ RASCEU RAWDOI RESORA03 RESORA13 SAGSOJ SIJHOJ SOPXUQ SUZXAN UCOFIC UDEMAR UKILAC UKILAC01 VOGRUE WAJJOG01 WANNII WETFAD01 WIKFIF WIZNAV XAXSIY YOTXUB ZZZGWU01

Phenol molecules (retained) with *E*(O,Ho)=0

ACAYIN AKUSEE AKUSII BERKEQ BIPXEF DPPHOL01 EFEMOT FECYUI GANVAS JABVUD KANJUF KAZHAU LIDJAK MBPHOL01 TUJYAZ XILZEX XMBPPR

Phenol molecules discarded, due to wrong hydroxyl orientation.

KAZHAU

LIST of alcohols-phenols

EHAVAL ENOJOH ENOKAU EWUGUY FEGFAZ GAYKEW GOLMOL IQESEC IQESIG IQESOM JAGQOX KEKTIE KOHJOG NURXED SAWHON SIPROY TITHOU UPAHUP XIJFEB XURHUO XUYRUF

Alcohol-phenol molecule discarded

MERHIB

LIST of hydroxyl-carbonyls

ATEPOE BALGAW BASYID BERJIR BEXGEQ BOBVIZ BOKVUS BOVMUU CUCHOX DAWQUO DESVIG DHPRTO02 DICREM DIYMOO DUNHOK DUZPUJ DXTMPO EADSUX EFEPOW EGUCOA EHALUU EHNPRG EJABUN ELOPUQ EYNAPO10 FAFSOV FAGYER01 FAVBAH FICPOY FITYUC FIZLIK FUXMOA01 GELQOD GERRAX GIVROT GODWAZ HIHTIB HIRROQ HMEPRG HMMAZO HMNPRG HNOBCH HONSUY HPRGDO HTCDOD HXPRED ICUQAZI CUQAZ01 IQOCUM IVOFUU JAZCIX JIWPEL JIWPEL01 JOHMOK KEFCED KIFGIP KUCDER KUMZEX LAVJAT LESNEE LIQCEU LIXFIH LIXFON LIXFUT LOBPID LOCJOD LOFZIQ LOSKAG MERBUH MILVUZ MILWAG MIMREG MTHPRG NAMMU KNETIN DNETIND01 NETIND02 NORGES01 NORGES10 NUBYUF OHMEDC10 OHUNEL PAGLEO PEZFEG01 PIHHAR POKQOX POSJAI PRGDOL02 PUVLOI QAQVUA QEQMII QEXLUB QINFOI QUYTUA RIHJEY RIMQUB RONTET SEBNUJ SEHCAK SIYWON TANBEP TEGWAD TETNUB TETPAJ TETPEN TIFNIG TIFNUS TIMVAM TOLNEO TOLNIS TONWIE UKUBAD ULEMEE UQOHOY URAJON UZEVEB VABQAS VEXKEO VIDVIP VIHCAR VIHCEV VOHTAP WAMVIQ WAVWOF WAVXIA WAVYAT WEFWEL WELXIU WEQMAG WOBKAZ YEGZOZ YOFWOG ZUDQAQ

LIST of hydroxyl-carbonyls dimers

BARYAU EGUCUG EJAYEU EYHENO EZOQUF GAHJEE KESKAV MACKAD1 NOLFIF RAKZOT RHODON SOFJEC VAPROU XETXIF XMPRYO YABCEJ ZZZPNG01

LIST of alcohols-monohydrate

BABYUA COQCOA ENIVON ESEDMH EVARUP FOZWEX GATVED GIXFID GIYJAA HXVITD IRIDE JAPZAB MYTOLD01 NAYKUT PECXIE TAGSAV01 TILZUL TIVBOP XAXKEO

Structure discarded : UBUGON

LISTof alcohols-ketones-monohydrate

ACOWAQ BEBFOD BOQSUV CAMTUG EGOTOL GOFCUB ITEJEW JIFYEC KEFCIH LIBWUQ NIFFEN NUSPEX PIRBUP SEZBAB TOVTED UQABIY VIDVAH VOQWEE WINLIP XABMAQ XEPGAC YAFQIG YAHQED

Structures discarded: ITEJEW YAHQED

LIST of chloro-ethers

AXAGUC AZOPEL BONDEP EDIZEW FAMFOO FUDBAI FUYJUF HEWJIE HUGBAN IDEFIG JEHJOV LINPIH LOCBEM MERQIK MERQOQ MERQUW MERRAD MERREH MILPUT MONGAY MUWROM NAVGAU ODOTUW ODOVEI OSOKEN PODJIC PUYSIL REBKOY TITQAO UDANAP VAFBEJ WINRUG YAWLIT

LIST of chloro-ketones

ABEFOD ACOQIU AGAQUW AGOLEN AXAREX AZIBER BAGZIS BAMYUJ BEMMEL BIMVAV BIMVEZ BOKJIW BZQDCL10 BZQDCL11 CBCYHO CEKZEX CIGGUU CIQFAK CLBENQ CLMANO CLPMCY01 CLTCOT CODHAE CORCUH CORDAO COXBST CPCPRO10 DALKIM DASMIU DASNOA DCLBQN DCLBQN01 DCLBQN03 DCLBZQ20 DIKYUR DISWEJ DISZIO DITYAG DITYAG10 DIYYUG DOXQIQ DUJPUU DUVFOP20 DUXTUL DUZSIA DUZSUM DUZTAT DUZTIB EBEQOR ECECUL EFUFER EKUYUF ELEWEX EROFAT EVIKAW EXODAW EXODIE EXODOK EYISIO FABGUK FABRAB10 FABRAB11 FAKKAD FAKKEH FAKKOR FOMSAB FUWNAM GATRAU GAVBEL GICMUA GIHGAG GIKNUJ HACJOK HENBEH HILFUF HIZGOO HOQTAJ HUDHAQ HUFZAK HUGQOQ IBEPEL IDIRIY IFINES IFORIG IJOWUA INEHAK IPUVOF JEQVUX01 JIZQEQ JIZQEQ01 JORGEC JORGIG KAFXIY KAVDOA KAVDUG KIDBUU KOSKIM KUMKUY KUMKUY10 KUNRAM LAPGIS LAPHAL LAPROL LEBGUU LEGXOM LEJDAH LEJDEL LEJDIP LEJDOV LEJYUU LUGMUW MATDUG MEGHUD MEHGAI MIGXAC MIYCED MIYLAH MODSUU MUCONC10 MUDBUJ MUDREJ MUQZED NAFCAY NAFQEQ NORBAY OGOROR OHIBOW02 OKEPUP OMIVAH OMIZUF PABZAT PAMPEY PAQJUO PEJHOD PEJHOD01 PEJJOF PEJJOF01 PEJKAS PEKDUF PIQLOQ PIQSEN PIRDEB PIWRES POJWUG POJXAN PONPEO PUKJEL PUQSOJ PUYWUB QAVGID QEQYAN QEWXIA QEWXIA01 QIQBEZ QOWNAR RISHOR ROFVIS ROGPEK ROMVEV RUFGOP RUPSIE SENZAO SIMTUE SUQDAJ SUQFAL SUQFIT SUQGEQ TAJBUB TAWLAE TCOXHN TIFZOX TIGSAE TIMNUA TIVBEG TOMZIG TURBAK UBIKEV UCOXUG UNUVAB UREKEI UVEKOW VEDXEH VIDHOF VIKLIK VUKVEC WAHNOI WAKWOW WEDMOH WIRCUV WOBBUL WOJGOS XEGLEA XEZHEP XEZNEX XUGVOK XUGYAZ XUGZEE YAKHOH YIFDUN YIVGIU YUDGAG ZEPVOH ZOBVAN

LIST of chloro-alcohols

AKEKEH ANALEH CUGPEA CUGPIE DADZAK DADZIS DAWMOE EJIWOJ EXOPEM GAKNAH HHCLMI HOYCOO HOYCUU ICUREE ICUROO ICUSAB ICUSOP IDOGEN IDURII IKUYUJ KIRBAQ LAKFOS LEPWUY LESPOQ MUDZUH NESVAJ NETBAQ OCENOA OCENUG QEVRIT QEWYUN RAJFIT SIFXEL TEFSAA TIMROW TIMRUC UFEQON ULEPOR VIWNAS WECPEB WECPIF WEGGOF WEGHAS WIXYEI XEHKIE XEHLEB01 XULSOM

Table Sup 2.

Statistics on *E* and *E*’ values for all families of compounds.

Definition used here for “Mean value E” : <E> = <E’> if <E’> is smaller than 1.
<E> = 2 – 1 / <E’> if <E’> > is larger than 1.

ssd E' : sample standard deviation
Std err Mean E' : Standard deviation of Mean E’
No. structures : Number of structures

Ketones

Contact	H...H	O...H	O...O
Mean value *E'*	0,97	1,14	0,02
Mean value *E*	0,97	1,16	0.02
ssd *E'*	0,04	0,08	0,07
Std err Mean *E'*	0,004	0,01	0,01
Std err Mean *E*	0,004	0,01	0,01
No. structures	119		

Ethers

Contact	H...H	C...H	O...H
Mean value E'	0,97	1,04	1,12
Mean value E	0,97	1,05	1,14
ssd E’	0,04	0,08	0,09
Std err Mean E'	0,01	0,01	0,01
Std err Mean E	0,01	0,01	0,01
No. structures	39		

Nitro-Ethers

Contact	H...H	H...Occ	H...On
Mean value E'	0,84	1,03	1,31
Mean value E	0,86	1,03	1,45
Ssd E’	0,23	0,49	0,11
Std err Mean E'	0,05	0,10	0,02
Std err Mean E	0,05	0,10	0,02
No. structures	24		

Esters

Contact	H...H	H...Occ	H...O=c
Mean value E'	0,92	1,07	1,19
Mean value E	0,93	1,07	1,23
ssd E’	0,07	0,12	0,10
Std err Mean E'	0,01	0,01	0,01
No. structures	123		

Alcohols

Contact	O...Ho	O...O	C...O	Hc...Ho	O...Hc	Hc...Hc	C...Hc
Mean value E'	1,80	0,10	0,25	0,54	0,56	1,09	1,11
Mean value E	5,08	0,10	0,25	0,54	0,56	1,10	1,13
ssd E’	0,11	0,37	0,30	0,16	0,16	0,06	0,14
Std err Mean E'	0,01	0,03	0,03	0,01	0,01	0,00	0,01
No. structures	144						

Phenols

Contact	Hc...Ho	O...Hc	Hc...Hc	C...O	C...Hc	O...Ho	O...O
Mean value E'	0,60	0,88	0,92	0,37	1,14	1,41	0,17
Mean value E	0,60	0,88	0,92	0,37	1,17	1,69	0,17
ssd E’	0,29	0,28	0,22	0,34	0,16	0,76	0,56
Std err Mean E'	0,03	0,03	0,02	0,04	0,02	0,08	0,06
No. structures	81						

Alcohol-Phenol

Contact	C...Ha	C...Hp	C...C	Hc...Ha	Hc...Hp	Hc...Oa	Hc...Op
Mean value E'	0,81	0,22	0,69	0,64	0,69	0,61	0,94
Mean value E	0,81	0,22	0,69	0,64	0,69	0,61	0,94
ssd E’	0,61	0,32	0,33	0,32	0,30	0,28	0,25
Std err Mean E'	0,14	0,07	0,07	0,07	0,07	0,06	0,06
No. structures	20						

Contact	C...Hc	Hc...Hc	Hp...Oa	Hp...Op	Ha...Oa	Ha...Op
Mean value E'	1,15	1,00	1,63	0,18	0,90	1,20
Mean value E	1,18	1,00	2,69	0,18	0,90	1,25
ssd E’	0,10	0,08	0,70	0,54	0,78	0,91
Std err Mean E'	0,02	0,02	0,16	0,12	0,17	0,20

Alcohol-ketone

Contact	Hc...Ha	Hc...Hc	Hc...Oa	Hc...O=c	C...Hc	Ha...Oa	Ha...O=c	C...Ha
Mean value E'	0,65	0,99	1,08	0,96	1,06	0,43	1,55	0,50
Mean value E	0,65	0,99	1,08	0,96	1,07	0,43	2,22	0,50
ssd E'	0,23	0,06	0,24	0,22	0,11	0,73	0,64	0,44
Std err Mean E'	0,02	0,01	0,02	0,02	0,01	0,06	0,06	0,04
No. structures	133							

Alcohol monohydrate

contact	Oa…Ha	Ow…Ha	Oa…Hw	Ow…Hw	Oa…Hc	Ow…Hc
Mean value E'	1,32	1,80	1,75	0,40	0,46	0,61
Mean value E	1,47	5,12	3,99	0,40	0,46	0,61
ssd E'	0,70	0,12	0,17	0,66	0,25	0,28
Std err Mean E'	0,16	0,03	0,04	0,15	0,06	0,07
No. structures	19					

contact	Hc…C	Hc…Hc	Hw…Ha	Hc…Ha	Hw…Hw	Hw...Hc	Ha...Ha
Mean value E'	1,14	1,15	1,04	0,42	0,48	0,69	0,78
Mean value E	1,16	1,17	1,04	0,42	0,48	0,69	0,78
ssd E'	0,26	0,14	0,50	0,21	0,51	0,16	0,52
Std err Mean E'	0,06	0,03	0,11	0,05	0,12	0,04	0,12

Alcohol-ketone monohydrate

Contact	Ha…Oa	Hw…Oa	Ha…Ow	Hw...Ow	Ha…O=c	Hw…O=c
Mean value E'	0,59	1,48	1,86	0,13	0,53	1,46
Mean value E	0,71	1,93	7,01	0,53	0,68	1,84
ssd E’	0,81	0,62	0,05	0,39	0,81	0,60
Std err Mean E'	0,17	0,13	0,01	0,08	0,17	0,13
No. structures	22					

Contact	Hc...O=c	Hc...Oa	Hc…Ow
Mean value E'	0,94	0,81	0,82
Mean value E	0,95	0,84	0,85
ssd E’	0,23	0,37	0,27
Std err Mean E'	0,05	0,08	0,06

Chloro-ether

Contact	Cl…Cl	Cl…Hc	Hc…Hc	C…Cl	C…Hc	C…C	O…Cl	O…Hc
Mean value E'	0,55	1,17	0,84	0,95	1,00	0,77	0,40	1,24
Mean value E	0,69	1,20	0,86	0,95	1,00	0,81	0,62	1,32
ssd E’	0,44	0,10	0,10	0,35	0,25	0,44	0,46	0,27
Std err Mean E'	0,08	0,02	0,02	0,06	0,04	0,08	0,08	0,05
No. structures	33							

Contact	O…C	O…O
Mean value E'	0,35	0,11
Mean value E	0,61	0,53
ssd E’	0,43	0,35
Std err Mean E'	0,08	0,06

Chloro ketones

Contact	Cl…Cl	Cl…H	H…H	C…Cl	C…H	C…C	O…Cl	O…H
Mean value E'	0,64	1,16	0,78	0,87	0,89	0,99	0,44	1,36
Mean value E	0,74	1,19	0,82	0,88	0,90	0,99	0,64	1,55
ssd E’	0,51	0,20	0,23	0,34	0,27	0,44	0,45	0,16
Std err Mean E'	0,04	0,01	0,02	0,02	0,02	0,03	0,03	0,01
No. structures	203							

Contact	O…C	O…O
Mean value E'	0,63	0,06
Mean value E	0,73	0,52
ssd E’	0,52	0,20
Std err Mean E'	0,04	0,01

Chloro-alcohols

Contact	Cl…Cl	Cl…Ho	Ho…Ho	C…Cl	C…Ho	C…C	O…Cl	O…C
Mean value E'	0,56	0,55	0,49	0,85	0,64	0,82	0,17	0,63
Mean value E	0,70	0,69	0,66	0,87	0,73	0,85	0,55	0,73
ssd E’	0,53	0,62	0,60	0,36	0,54	0,49	0,26	2,67
Std err Mean E'	0,08	0,09	0,09	0,05	0,08	0,07	0,04	0,40
No. structures	45							

Contact	O…Ho	O…O	Cl…Hc	Hc…Ho	C…Hc	O…Hc	Hc…Hc
Mean value E'	1,48	0,06	1,18	0,60	1,07	0,90	0,75
Mean value E	1,91	0,52	1,21	0,71	1,08	0,91	0,80
ssd E’	0,75	0,27	0,23	0,28	0,25	0,43	0,29
Std err Mean E'	0,11	0,04	0,03	0,04	0,04	0,06	0,04

Protocol to search molecules in the CSD.

To find molecules in the CSD , the following options were always applied:

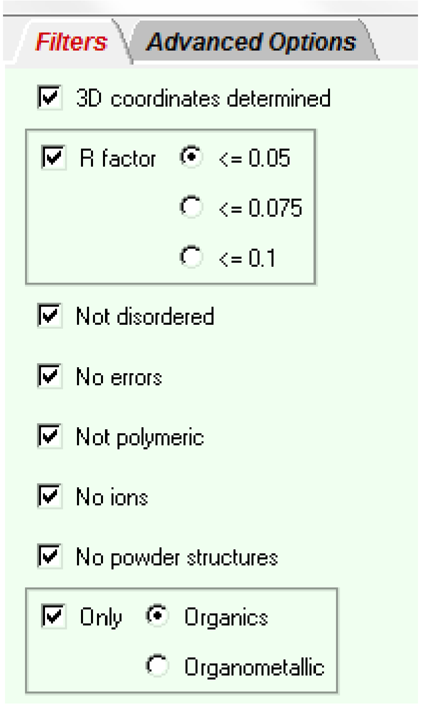

To search, for example, the chloro-ether molecules in the CSD with Conquest, the following steps were applied:

1) Molecules containing C H O Cl were searched
2) Molecules containing an ether C-O-C group were searched
3) Molecules containing a O-H group were searched
4) Molecules containing a C=O group were searched
5) A combination of : must have (1) & (2) must not have (3) & (4) was done
6) Remaining molecules were inspected on screen and selected if suitable. Molecules with missing hydrogen, errors, with solvent molecules or multimers were discarded.
